# Heat shock protein–mediated remodeling of the bone immune microenvironment: mechanisms and precision therapeutic strategies for osteoporosis

**DOI:** 10.1186/s12967-026-08389-3

**Published:** 2026-06-08

**Authors:** Jinyang An, Jia Bai, Lingling Li, Xinsai Li, Yangyang Zhang, Haihong Lv

**Affiliations:** 1 The First Clinical Medical College of Lanzhou University, Lanzhou, Gansu 730000 China; 2 Department of Endocrinology, The First Hospital of Lanzhou University, No. 1 West Donggang Road, Lanzhou, Gansu 730000 China; 3 Gansu Clinical Medical Research Center for Endocrine Diseases, Gansu, 730000 China

**Keywords:** Heat shock proteins, Osteoporosis, Bone immunology, Homeostasis regulation, Prediction model, Personalized regulation

## Abstract

**Background:**

Osteoporosis is a systemic bone disorder marked by reduced bone mass and deteriorating bone microstructure, commonly associated with inflammation, oxidative stress, and imbalances in immune homeostasis. Among the key mechanisms underlying Osteoporosis progression, disruption of the bone immune microenvironment has emerged as a critical scientific issue. Heat shock proteins (HSPs), as highly conserved molecular chaperones and stress-responsive proteins, not only prevent protein misfolding and aggregation under stress conditions to maintain protein homeostasis, but also participate in immune signaling and adaptive stress responses, thereby playing important roles in preserving bone metabolic homeostasis.

**Main body of the abstract:**

This review aims to systematically examine the roles of HSP families—HSPB, HSP40, HSP70, HSP90, and HSP110—in bone remodeling, the balance between osteogenesis and osteoclastogenesis, and the interaction between endoplasmic reticulum stress and mitochondrial energy metabolism. The review also highlights the dual roles of HSPs in regulating bone immune homeostasis. HSPs participate at the cellular level in regulating osteoblast, osteoclast, and bone marrow mesenchymal stem cell function. Current evidence suggests that HSPs act as both molecular chaperones and stress-responsive immune modulators, regulating the balance between bone formation and bone resorption and remodeling the bone immune microenvironment. This review also summarizes HSP-targeted therapeutic strategies, including small molecules, natural products, neutralizing antibodies, physical stimulation, and gene- and cell-based therapies.

**Conclusion:**

Research on HSPs is shifting from molecular mechanisms toward precision intervention. Future studies should integrate the “bone-muscle-immune axis” with artificial intelligence and multi-omics technologies to construct a “heat shock response-bone immune” network, identify novel therapeutic targets, and promote personalized prevention and treatment of osteoporosis.

## Background

Osteoporosis (OP), often referred to as the “silent bone thief,” is a systemic skeletal disease characterized by decreased bone mass and disruption of bone tissue microstructure, leading to increased bone fragility and an elevated risk of fractures. This chronic condition is closely associated with fractures and even mortality in the elderly, imposing a significant burden on families and society [1]. The pathogenesis of osteoporosis is complex and is often the result of multiple factors interacting, with research spanning multiple disciplines. Scholars have developed various theories from different perspectives, including the theory of bone metabolism imbalance [2], angiogenesis and osteoporosis theory [3], endocrine theory (estrogen deficiency) [4], oxidative stress theory [5], biomechanics theory [6], and traditional Chinese medicine theory [7]. It was previously believed that bones functioned as a relatively independent system; however, increasing evidence shows that the immune system and bones are closely interconnected, sharing molecular regulatory networks and microenvironments (such as bone marrow) [8]. The two systems influence each other through cytokines, signaling pathways, and immune cell activity [9]. This interdisciplinary research field, which bridges immunology and bone biology, is referred to as “bone immunology.” Bone immunology originated from the study of the inflammatory mediators secreted by leukocytes and their interaction with osteoclasts (OCs) [10]. Subsequently, the regulatory relationships between immune factors and osteoblasts (OBs) [11], bone marrow mesenchymal stem cells (BMSCs) [12], and other bone cells have been gradually elucidated.

Bone remodeling is a dynamic, continuous process in which OBs mediate bone formation, OCs mediate bone resorption, and osteocytes regulate the formation–resorption balance [13]. Osteocytes are the most abundant cell type in the skeleton, accounting for greater than 90% of all bone cells [14]. After completing their bone-forming function, OBs become encased in the matrix they secrete and, upon subsequent mineralization, differentiate into osteocytes [15]. Although osteocytes play crucial roles in skeletal maintenance, regulation, and mechanosensation [16], they are often regarded as relatively quiescent compared with the functionally active OBs, OCs, and BMSCs. Evidence indicates that cortical bone, where osteocytes are the predominant cellular component, remodels at an annual rate of approximately 3.3%, whereas trabecular bone (the principal component of the cancellous compartment) remodels at approximately 14.3% per year [17]. Because OBs and OCs—the major cellular constituents of trabecular bone—exhibit relatively higher activity, imbalances in bone turnover are more pronounced in this compartment under conditions such as aging and disease [18, 19].

BMSCs account for only 0.01% to 0.001% of the total cell population in bone marrow [20], yet they possess the capacity to differentiate into OBs, chondrocytes (CCs), and adipocytes, playing a crucial role in skeletal development, homeostasis, and repair [21]. Under physiological conditions, BMSCs predominantly differentiate into osteoblasts; however, under pathological conditions such as aging, estrogen deficiency, or inflammatory stress, BMSCs are redirected toward adipogenic differentiation [22]. Moreover, these cells exhibit significant immunomodulatory properties, capable of suppressing excessive inflammatory responses and improving the inflammatory microenvironment to support bone repair [23]. BMSCs serve as both the cellular source of bone formation and as mediators of the immune-skeletal dialogue, playing a pivotal role in bone immunology by regulating differentiation potential and immune function.

Cartilage lacks blood vessels, lymphatic vessels, and nerve supply, and its resident cells have limited proliferative capacity, which has traditionally led to the view that cartilage is an immune-privileged tissue [24]. Although the strength and diversity of the immune response in chondrocytes may still be limited compared to immune cells in vascularized tissues, recent studies have uncovered an active role of chondrocytes in inflammatory responses, as well as the critical role of damage-associated molecular patterns (DAMPs) in this process [25, 26]. The bone microenvironment is a multidimensional functional system continuously remodeled and constructed by BMSCs, OBs, osteocytes, OCs, immune cells, and endothelial cells. The development and lifelong bone remodeling process rely on the coordinated action of these cells at every stage [27].

In general, immune imbalance initially triggers inflammation and oxidative stress, driving bone resorption and inducing OP. This process is highly complex, but under coordinated regulation by the organism, bone loss can be alleviated or even reversed. TNF-α at low concentrations or short-term exposure can promote the osteogenic differentiation of BMSCs, while high concentrations or prolonged exposure activate osteoclastogenesis, exhibiting a typical biphasic curve [28], Other inflammatory factors, such as IFN-γ, can also promote the osteogenic differentiation of BMSCs [29]. Subsequent studies have shown that, in order to maintain homeostasis and self-protection, the body secretes various substances, including heat shock proteins (HSPs), to enhance cellular adaptability [30].

**Fig. 1:**
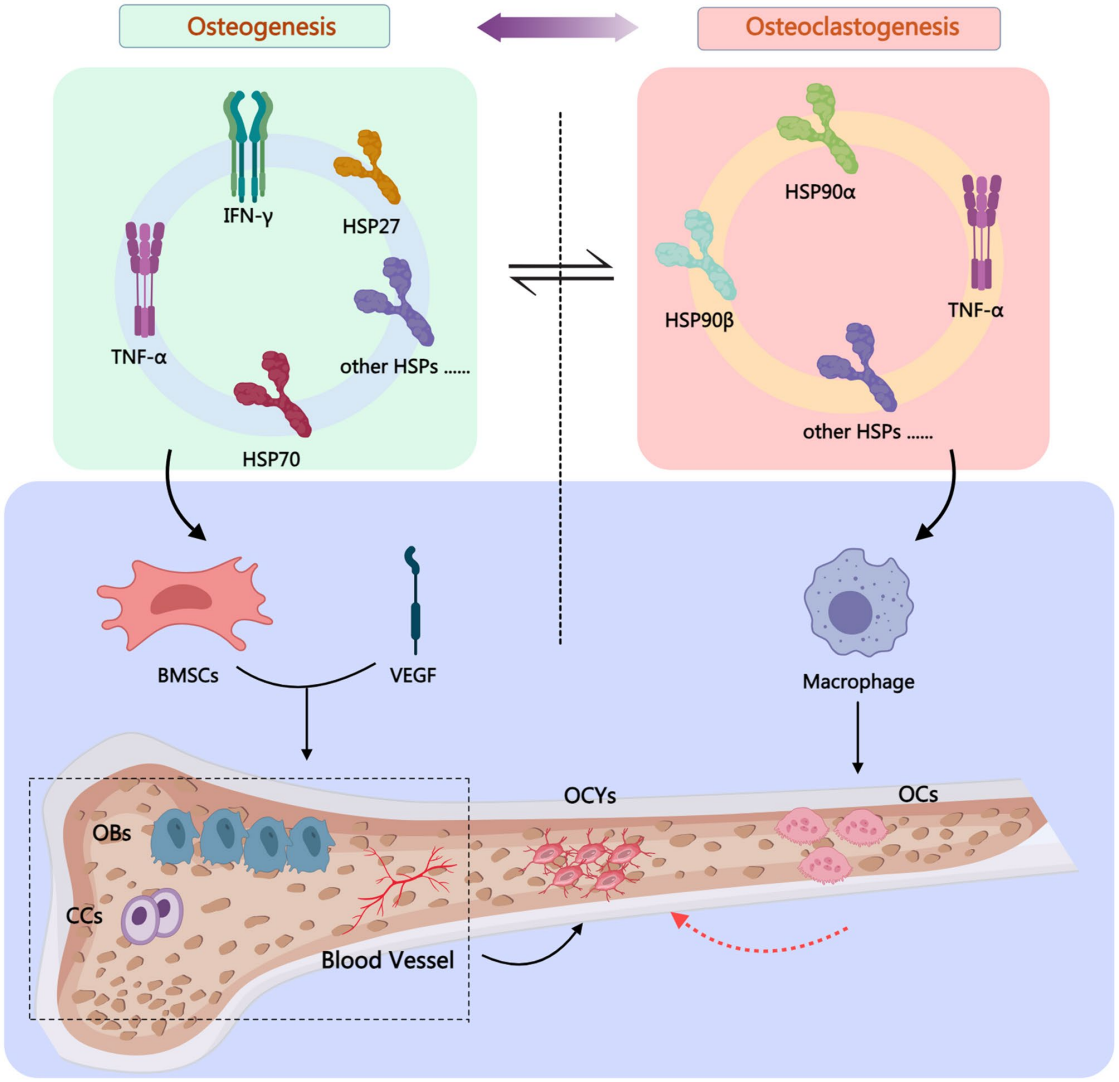
Schematic representation of the regulatory role of heat shock proteins in osteogenic and osteoclastic differentiation in bone metabolism from the perspective of bone immunology. Bone loss in the bone microenvironment is closely related to the imbalance of the immune-bone metabolism network. This figure summarizes the key regulatory roles of some representative members of the HSPs family in the differentiation processes of OBs and OCs from the perspective of overall bone immunology regulation. On the left, during the osteogenesis process, HSP27, HSP70, and others promote the osteogenic differentiation of BMSCs, induce the release of VEGF, and enhance angiogenesis, working together with immune factors such as IFN-γ and TNF-α to maintain bone formation. On the right, during the osteoclastogenesis process, upregulation of HSP90 family-related proteins drives the differentiation of macrophages into mature OCs, with TNF-α also playing a synergistic role in enhancing bone resorption. As core mediators of cellular stress and homeostasis regulation, HSPs exert bidirectional regulatory functions under pathological conditions such as inflammation and oxidative stress. The expression balance of their family members profoundly influences bone metabolism outcomes, providing important insights for understanding the mechanisms of osteoporosis and developing targeted therapeutic strategies. BMSCs: bone marrow mesenchymal stem cells; CCs: chondrocytes; OBs: osteoblasts; OCs: osteoclasts; OCYs: Osteocytes; VEGF:Vascular Endothelial Growth Factor (the figure was created in Medpeer)

Typically, HSPs play a crucial role in preventing excessive inflammatory responses and facilitating the correct folding and reorganization of intracellular proteins [31]. When HSPs are dysfunctional, it leads to an increased rate of protein misfolding and the accumulation of toxic substances in subcellular regions, which are difficult to clear [32]. As shown in Fig. 1, the HSP family, as key regulators of homeostasis, consists of numerous members with diverse functions, playing a vital role in determining the outcomes of bone metabolism. For example, HSP27 can co-localize with the cytoskeleton and promote osteogenic differentiation in BMSCs [33]. HSP70 directly participates in angiogenesis by regulating endothelial cell functions such as migration and vessel formation [34], while HSP27 also plays a role in TGF-β induced VEGF release [35], further promoting osteogenic differentiation of BMSCs [36]. HSP90α plays a critical role in the differentiation, formation, and activation of OCs, with its expression significantly upregulated during the differentiation of OC precursors into mature OCs [37].

We incorporated HSPs into the osteoimmunology framework, emphasizing their dual functions as intracellular chaperones that preserve bone cell homeostasis and as extracellular stress signals that regulate immune-mediated bone remodeling. We further proposed a multilevel intervention strategy that integrates multiple research paradigms and advocates the use of advanced approaches, including artificial intelligence-driven multi-omics, to characterize the “heat shock response-osteoimmunology” network and identify novel therapeutic targets. Overall, this HSP-centered perspective clarifies key mechanisms underlying osteoporosis, provides a robust scientific basis for precision intervention, and highlights its translational potential for future therapeutic development.

## Main text

### Overview and function of the HSP family

Research on the HSPs family has spanned over 60 years, with their discovery marking a milestone event in 20th-century biology. Professor Ritossa inadvertently increased the temperature of the medium from the normal 25 °C to 30 °C while culturing fruit fly larvae. Within a short period of temperature elevation, specific “puffing” patterns appeared on the chromosomes of the fruit fly salivary glands. He realized that this phenomenon could be related to the activation of certain genes and the subsequent increase in protein synthesis under heat stress [38]. Subsequent studies confirmed that the “puffing” phenomenon observed by Ritossa indeed corresponded to the synthesis of a series of new proteins [39]. These proteins, induced under heat stress conditions, were later collectively termed HSPs. The induced expression of HSPs is not confined to heat stress alone. A variety of stress conditions, including hypoxia, ischemia, cold, heavy metal exposure, viral infections, and inflammation factor stimulation, can trigger the upregulation of HSPs expression. As a result, HSPs are more broadly referred to as “stress proteins” [40]. Although the term “stress proteins” more comprehensively encompasses the diverse induction conditions, the initial designation of HSPs has been retained due to its widespread recognition.

With the advancement of research, numerous HSPs have been identified. However, the lack of a unified naming convention has led to confusion in their classification. In 2009, Hageman J and his team systematically reviewed various heat shock protein families, specifically including HSPH (HSP110), HSPC (HSP90), HSPA (HSP70), DNAJ (HSP40), and HSPB (sHSPs), terms that have since been standardized by the Human Gene Nomenclature Committee [41]. Each family contains multiple members, which may have different subcellular localizations (such as cytoplasm, nucleus, mitochondria, and endoplasmic reticulum (ER)) and distinct substrate-binding characteristics, thus participating in the regulation of various cellular processes [42]. Members of the HSP90 family typically have molecular weights ranging from 80 to 94 kDa, while those of the HSP70 family range from 66 to 78 kDa. sHSPs, with relatively smaller molecular weights, generally fall between 12 and 43 kDa. This molecular weight-based classification provides researchers with a preliminary framework to distinguish and study the functions of different HSPs [42].

The expression of HSPs is primarily regulated at the transcriptional level, with heat shock transcription factors (HSFs) playing a central role in this process. HSFs are a class of proteins that specifically bind to heat shock elements (HSEs) in the promoter regions of HSP genes, thereby activating the transcription of HSP genes [43]. In mammals, multiple HSF family members exist (HSF1, HSF2, HSF3, HSF4, HSFY, etc.), with HSF1 being considered the most important transcription factor mediating the classic heat shock response (HSR) and inducing the expression of HSPs [44]. Under normal physiological conditions, HSF1 exists in an inactive monomeric form in the cytoplasm or bound to molecular chaperones (such as HSP70 and HSP90), maintaining its inactive state and preventing activation, nuclear localization, and DNA binding. When cells are subjected to heat shock or other stress stimuli, the accumulation of misfolded proteins competitively binds to molecular chaperones, leading to the release of HSF1 [45]. The activity of HSF1 is regulated by various mechanisms, including negative feedback. Upon the increased expression of HSPs, they bind to HSF1, promoting its dissociation from DNA and inactivating it, thereby halting HSP transcription and ensuring that expression returns to baseline levels once the stress is resolved [40, 46].

The core function of HSPs is to act as molecular chaperones, participating in the maintenance of protein homeostasis within the cell. They assist other proteins in achieving the correct three-dimensional folding, assembling into functional complexes, and facilitating their transport across different cellular compartments through various mechanisms [47]. HSPs and HSFs play a critical role not only in the stress response but also in the regulation of various cellular differentiation processes. For instance, in the hematopoietic system, HSFs and HSPs regulate the differentiation of various hematopoietic-derived cells, such as erythrocytes, macrophages, B cells, and T cells [48]. HSP60 promotes osteogenesis and alleviates glucocorticoid-induced osteoporosis by stabilizing Regulatory-associated protein of mTOR, activating osteocytic autophagy, and protecting cells from apoptosis [49]. HSP90β, by stabilizing the protein levels of the core kinase LATS in the Hippo signaling pathway, inhibits its activity and activates the YAP protein, thereby promoting osteoblastic differentiation [50]. Although most HSPs facilitate osteogenesis, certain members of this family, such as HSP90α, have a negative regulatory effect on bone metabolism [37].

HSPs, as molecular chaperones and immune modulators, play a pivotal role in the regulatory network of bone remodeling. On one hand, they directly influence the differentiation and function of OBs, OCs, and BMSCs by maintaining protein homeostasis within these cells. On the other hand, they indirectly regulate the balance of the bone immune microenvironment by suppressing excessive inflammatory responses and oxidative stress. Integrating HSPs into the research framework of bone immunology offers the potential to elucidate novel molecular mechanisms underlying the pathogenesis of OP. Furthermore, it provides a theoretical basis for targeted interventions aimed at the HSF/HSP axis or protein homeostasis networks, ultimately improving bone quality and reducing fracture risk in patients.

#### Heat shock proteins family and bone metabolism

##### The HSPB (sHSP) family and bone metabolism

The HSPB (sHSP) family in the human genome consists of 11 members, with HSPB1 being the most extensively studied representative, classified as a stress-induced protein. Additionally, HSPB4 and HSPB5 are highly associated with diseases in multiple tissues, including muscles, the heart, and the lens [51]. The interaction of HSPB1 with components of the cytoskeleton helps maintain the structural integrity and functional activity of the cytoskeleton under stress conditions [52]. As non-ATP-dependent chaperones, sHSPs play a crucial role as the “first line of defense” in cellular stress responses. They recognize and bind to unfolded or misfolded proteins, preventing their irreversible aggregation, thereby stabilizing the intracellular environment. This non-ATP-dependent binding capacity allows sHSPs to remain effective under stress conditions where ATP levels are compromised (e.g., energy depletion) [53].

Members of the sHSP family primarily participate in regulating the dynamic balance between bone formation and bone resorption by influencing the differentiation, activity, migration, and cytokine secretion of OBs and OCs. Specific members, such as HSPB1, HSPB5, and HSPB7, play significant roles in bone development, bone repair, and the pathological processes of OP through various molecular mechanisms, including phosphorylation modifications, regulation of the Wnt/β-catenin signaling pathway, inhibition of ferroptosis, and modulation of the RANKL/OPG system.

#### HSPB1 (HSP27) and bone metabolism

##### Overview of HSPB1

HSPB1 is a stress-inducible, ATP-independent sHSP that contributes to cytoprotection across disease and aging contexts by sequestering misfolded proteins, inhibiting apoptosis, and stabilizing the cytoskeleton [54]. he N-terminal region of HSPB1 contains three conserved serine phosphorylation sites—Ser15, Ser78, and Ser82—which are rapidly phosphorylated by stress-activated kinases such as p38 MAPK under stress conditions. Phosphorylation shifts the oligomeric equilibrium, driving dissociation from large oligomers to smaller dimers and thereby activating its chaperone function to exert protective effects [55].

## HSPB1 and bone metabolism

In human bone marrow mesenchymal stem cells (hBMSCs), both lipofuscin, a marker of oxidative stress, and HSPB1 expression are upregulated under electric field stimulation. This is accompanied by a significant increase in the expression of osteogenic proteins, including alkaline phosphatase (ALP) and Collagen I (Col I). As a stress protein, HSPB1 may play a regulatory role in electromagnetic field-induced osteogenic differentiation, although the precise mechanism remains unclear [56]. Further studies have shown that pre-treatment with organic light-emitting diode red light enhances the survival and pro-angiogenic capabilities of hBMSCs. This effect is mediated by ROS-induced expression of HSP90α and HSPB1, activating the Akt-NF-κB pathway, thereby inhibiting apoptosis and promoting angiogenesis [57]. Given the critical role of HSPB1 in stress protection, researchers have also developed HSPB1 pre-treatment strategies, such as non-lethal heat stress, which significantly improve the anti-apoptotic capacity and survival rate of hBMSCs after bone marrow transplantation through time-dependent upregulation of HSPB1 expression [58]. In OB precursor cells MC3T3–E1, simvastatin treatment increases ALP activity and mineralized nodule formation, with mechanistic studies revealing that this effect is mediated by p38 MAPK-dependent specific upregulation of HSPB1 [59].

Upregulation of HSPB1 can inhibit the differentiation of bone marrow-derived macrophages into OCs. HSPB1 reduces ROS production and inhibits p38/AKT phosphorylation, thereby downregulating the expression of key genes involved in OC differentiation, such as c-fos and NFATc1. This ultimately decreases TRAP activity and bone resorption function [60]. In breast cancer cells, overexpression of HSPB1 reduces cell motility, and the number and volume of bone metastatic lesions are significantly reduced. These findings suggest that HSPB1 may be related to osteolytic activity, although the specific mechanism requires further investigation [61].

Phosphorylation of HSPB1 also plays a critical role in bone metabolism. Studies have shown that during the osteogenic differentiation of hBMSCs induced by bioactive glass, HSPB1 phosphorylation enables its interaction with the cytoskeletal protein F-actin, maintaining the mechanical microenvironment required for osteogenic precursor cells. Inhibition of HSPB1 phosphorylation results in the loss of cytoskeletal tension, leading to a significant decrease in ALP activity and mineralization levels [33]. This mechanism is particularly prominent under pathological conditions. In adolescent idiopathic scoliosis (AIS) patients, the phosphorylation level of HSPB1 is markedly reduced in OBs and BMSCs, which correlates directly with the decreased expression of bone formation markers (such as ALP, SPP1, and OCN) and impaired mineralization capacity. However, treatment with heat shock or the use of specific HSPB1 phosphorylation inducers significantly restores HSPB1 phosphorylation levels in AIS patient cells, thereby improving osteogenic function [62].

### HSPB5 (CRYAB, αB-crystallin) and bone metabolism

#### Overview of HSPB5

HSPB5, encoded by the *Cryab* gene, is a key member of the HSPB family. This protein is located on human chromosome 11q23.1 and is also known as CRYAB or αB-crystallin. Unlike other molecular chaperones, HSPB5 generally does not directly bind to target proteins but instead protects them from damage by forming oligomers that encapsulate the target proteins [63]. Initially discovered in the lens, HSPB5 is also expressed in various other tissues, including the heart, skeletal muscle, kidneys, lungs, astrocytes, and the brain [64]. HSPB5 is induced under stress conditions such as heat shock and hypertonicity, where it prevents protein aggregation [65]. Research has shown that HSPB5 targets inflammatory sites through temperature sensitivity and selective binding of pro-inflammatory proteins, offering localized regulation rather than systemic immune suppression. This selective targeting positions HSPB5 as a promising candidate for the development of precision anti-inflammatory therapies [66]. Additionally, HSPB5 can be transported between cells via exosomes, exhibiting advantages such as directionality, stability, and stress responsiveness, making it a potential candidate for clinical applications [67].

## HSPB5 and bone metabolism

Using proteomic techniques, including two-dimensional electrophoresis and mass spectrometry, researchers successfully identified HSPB5 in human demineralized bone powder and murine osteoblast precursor cells MC3T3–E1. Further analysis using immunofluorescence microscopy revealed that HSPB5 is expressed in the nucleus, cytoplasm, and cytoskeleton of osteoblast precursor cells, with its distribution pattern closely correlated with the cell proliferation state. It is hypothesized that HSPB5 may play a role in protecting the cytoskeleton, regulating transcription, apoptosis, and responding to stress [68].

Through whole-genome chip technology, *Cryab* was also found to be upregulated alongside genes associated with osteogenic differentiation in hBMSCs [69]. HSPB5 serves as a key positive regulator in osteogenic differentiation of hBMSCs. Overexpression of HSPB5 significantly enhances the osteogenic potential of hBMSCs, while knockdown of HSPB5 inhibits osteogenic differentiation and promotes adipogenesis. Mechanistic studies indicate that HSPB5 directly binds to β-catenin, preventing its ubiquitination and degradation, thereby upregulating the expression of osteogenic genes such as *Runt-related transcription factor 2* (*Runx2*) [70]. The mRNA levels of *Hspb5* in plasma from OP patients were significantly lower than in healthy controls. Research suggests that HSPB5 stabilizes FTH1 through a lactylation-dependent ubiquitin-proteasome pathway, inhibiting ferroptosis and maintaining the osteogenic differentiation potential of hBMSCs [71].

HSPB5 also has a potential positive regulatory role in cartilage development. Specifically, the early upregulation of HSPB5 in cartilage formation maintains the stability of the extracellular matrix structure, promoting the survival and differentiation of CC progenitors [72]. Furthermore, studies suggest that HSPB5 could be a potential therapeutic target for osteoarthritis. Downregulation of HSPB5 is negatively correlated with cartilage degeneration, while HSPB5 overexpression blocks apoptotic pathways, such as caspase-3 and Bax, helping to maintain the balance between cartilage extracellular matrix synthesis and degradation, and promoting cell proliferation and differentiation [73]. Proteomic analysis revealed that HSPB5 is significantly expressed and continuously upregulated during osteogenic differentiation of hBMSCs, whereas its expression remains almost unchanged during adipogenic and chondrogenic differentiation, positioning it as an “osteogenesis-specific” intracellular marker. Although previous studies indicated transient upregulation of HSPB5 in CC progenitors during early stages, in the hBMSC model, HSPB5 expression did not change, suggesting that its function may depend on cell type, species, or differentiation stage [74].

### HSPB7 and bone metabolism

#### Overview of HSPB7

HSPB7 is an important member of the small sHSP family. Studies have shown that HSPB7 can form dimers or large oligomers and interact with HSPB6 and HSPB8 to form heteromeric complexes. It may cooperate with these proteins in tissues such as muscle to exert molecular chaperone activity, antioxidant capacity, and cytoskeletal interactions [75].

## HSPB7 in bone metabolism

In BMSCs, HSPB7 acts as a negative regulator of osteogenic differentiation, and its expression is significantly downregulated during osteogenic induction. In BMSCs derived from OP mice, HSPB7 expression is upregulated, suggesting its involvement in the pathogenesis of osteoporosis. Studies have demonstrated that knockdown of HSPB7 enhances ALP activity, mineralized nodule formation, and osteogenic gene expression by activating the ERK signaling pathway, thereby promoting osteogenic differentiation. Conversely, overexpression of HSPB7 inhibits osteogenesis [76].

### Potential clinical value of the HSPB family in osteoporosis

From a translational perspective, the clinical relevance of the HSPB family extends beyond its role in regulating bone remodeling. Members of this family also show promise as sensitive biomarkers for OP. In patients with OP, plasma HSPB5 levels are significantly reduced [71], whereas HSPB7 expression is increased in BMSCs isolated from murine models of OP [76]. This functional heterogeneity within the HSPB family provides a useful basis for cross-validation in biomarker development.

Reduced HSPB5 expression may indicate impaired osteogenesis and increased marrow adiposity in patients with OP. Mechanistically, this association may be related to the role of HSPB5 in stabilizing β-catenin signaling. Thus, HSPB5 deficiency may define a subtype of OP characterized by impaired osteoblast differentiation and attenuated Wnt signaling [70, 71]. In such patients, the primary pathological abnormality is insufficient bone formation rather than excessive bone resorption. Consequently, anti-resorptive monotherapy may provide limited benefits in improving bone mineral density and reducing fracture risk, whereas combined or sequential anabolic therapies may be more appropriate. By contrast, elevated HSPB7 expression suggests that BMSCs retain a suppressed osteogenic phenotype, resulting in intrinsic inhibition of osteoblast activity [76]. In these patients, targeted strategies that relieve this inhibitory effect may improve prognosis. Therefore, measuring the expression of HSPB family members in blood or bone marrow-derived samples may support biomarker-based stratification, facilitate identification of distinct patient subgroups, and guide personalized treatment decisions.

In addition to their diagnostic potential, HSPB family members, particularly HSPB1, may serve as functional targets in regenerative medicine and cell-based therapy, thereby offering new therapeutic opportunities for OP. HSPB1 has considerable translational potential. It may be used as a pretreatment factor before stem cell transplantation to enhance the anti-apoptotic capacity of transplanted cells [56–58]. It may also be incorporated into bone repair materials as a functional molecule to optimize the local osteogenic microenvironment [33]. These applications may help overcome some limitations of conventional systemic drug delivery. In contrast, HSPB7 negatively regulates osteogenic differentiation, and its inhibition may relieve this suppressive effect and enhance bone formation. However, because HSPB7 is highly expressed in myocardial tissue and is essential for cardiac metabolic homeostasis [77, 78], strategies targeting HSPB7 should preferentially use local delivery approaches, such as intramedullary administration or gene silencing in transplanted cells, to minimize potential off-target effects in the heart and other high-expression tissues.

HSPB5 is a key positive regulator of osteogenic differentiation in hBMSCs [69, 70, 74]. Fusion of HSPB5 with the immunoglobulin Fc fragment has been reported to generate a secretable HSPB5-Fc fusion protein with cytoprotective effects in vitro, including prevention of harmful protein aggregation and stress-induced cellular injury [79]. This protein-engineering strategy provides a feasible approach for converting predominantly intracellular HSPB proteins into secreted therapeutic molecules. In the future, coupling engineered HSPB5 proteins with bone-targeted delivery systems may enable selective accumulation in bone tissue, thereby promoting bone repair and protecting osteoblasts. This strategy may represent a novel therapeutic avenue for OP.

Gene-regulatory therapies targeting the HSPB family are also being explored. Antisense oligonucleotides against HSPB1, such as apatorsen (OGX-427), have already entered oncology clinical trials and are used to suppress HSP27 expression and increase tumor sensitivity to treatment [80, 81]. Similarly, in OP, siRNA or antisense oligonucleotides could be used to inhibit HSPB members that impair osteogenesis, whereas gene delivery vectors could be used to overexpress beneficial family members and promote bone formation. However, these nucleic acid-based approaches require efficient bone-targeted delivery systems, such as nanocarriers or bone-affinitive liposomes, to reach the bone marrow microenvironment effectively [82, 83]. At present, this field remains in an early stage of development, and no mature HSPB-targeted gene therapies for OP have yet entered clinical trials.

Despite these advances, several major challenges must be addressed before HSPB-family research can be translated into effective clinical strategies for the prevention and treatment of OP. First, whether HSPB proteins are used as biomarkers or are assessed according to post-translational modifications, such as phosphorylation or lactylation, large-scale multicenter studies are needed to establish reference ranges and diagnostic thresholds for both healthy individuals and patients with OP. At present, most studies are exploratory and based on relatively small cohorts. For example, Daswani et al. reported a significant negative correlation between serum phosphorylated HSP27 and bone mineral density in perimenopausal women, providing preliminary support for its use as a biomarker; however, they also emphasized the need for validation in larger populations [84]. Second, the optimal timing of intervention and the most appropriate target populations remain unclear [84, 85]. If specific HSPB-related markers change significantly during the early stage of bone loss, it is important to determine whether interventions targeting these pathways should begin during osteopenia to prevent progression to OP. Conversely, in patients with established OP and high fracture risk, the magnitude of clinical benefit achievable through modulation of HSPB pathways remains uncertain. These questions require rigorous clinical investigation. In addition, population heterogeneity must be carefully considered. The biological roles of HSPB-related markers may vary according to age, sex, and menopausal status, and genetic background may also influence treatment response [86]. Accordingly, successful clinical translation will require a precision medicine framework, including subgroup analysis to identify the patients most likely to benefit and the development of individualized therapeutic strategies.

### HSP40 (DNAJ) family and bone metabolism

#### Overview of the HSP40 (DNAJ) family

The *Hsp40* gene is located on chromosome 19p13.12 of the human genome and contains eight exons. Proteins of the HSP40 family are characterized by a highly conserved C-terminal amino acid sequence known as the “J-domain” [87]. The chaperone activity of HSP40 requires the cooperation of HSP70. HSP40 first forms a complex with client proteins and then binds to HSP70 through its J-domain, thereby preventing protein misfolding and aggregation. This process is driven by ATP hydrolysis [88]. HSP40 precisely regulates the activation and function of HSP70 through class-specific autoinhibitory and release mechanisms-class A members efficiently assist in protein folding, whereas class B members possess a dual-site regulatory mechanism. This confers functional diversity upon the HSP40 family in various physiological processes, including protein folding and disaggregation [88].

#### HSP40 (DNAJ) and bone metabolism

However, current research on the relationship between HSP40 and bone metabolism remains limited. DNAJ, a major member of the HSP40 family, interacts with the ATPase domain of HSP70 through its J-domain, acting as a co-chaperone to form the “HSP40–HSP70-substrate” ternary complex. This interaction accelerates ATP hydrolysis, stabilizes folding intermediates, and prevents the aggregation of misfolded proteins [89]. The DNAJA3 (TID) splice variants TID-L and TID-I, both members of the HSP40 family, are upregulated during the osteogenic differentiation of hBMSCs, with expression peaking at later stages of differentiation. Further analysis showed that Tid gene silencing significantly altered SPP1 expression, suggesting that DNAJA3 may contribute to the molecular regulatory network governing osteogenic differentiation. Although in vivo and clinical evidence remains limited, these findings provide preliminary experimental support for the involvement of the HSP40 family in bone formation at the level of individual family members [90]. Luteolin selectively induces the expression of the HSP40 family member DNAJ. As a molecular co-chaperone, DNAJ directly binds to and destabilizes peroxisome proliferator–activated receptor γ (PPARγ), thereby inhibiting its transcriptional activity and suppressing adipogenesis. Concurrently, DNAJ forms a complex with Runx2, enhancing its DNA-binding capacity and promoting the expression of osteogenic genes such as *Alp* and *Osterix*. Through this HSP40-centered regulatory mechanism, DNAJ directs the lineage commitment of BMSCs toward osteogenesis rather than adipogenesis [91].

#### Potential clinical value of the HSP40 family in osteoporosis

Research on the role of HSP40 family proteins in bone metabolism is still in its early stages and remains limited by the lack of in vivo studies, clinical evidence, and validation in large cohorts. Nevertheless, the available findings suggest that the HSP40 family may have important clinical relevance in the regulation of bone metabolism. Studies showing that luteolin promotes osteogenesis through upregulation of DNAJ expression indicate that HSP40 acts not only at the terminal effector stage of bone metabolism but also at an upstream regulatory node that influences the osteogenic fate of BMSCs [91]. From a translational standpoint, such upstream regulatory nodes may represent promising targets for disease subtype stratification and early therapeutic intervention.

In addition, HSP40 functions intracellularly as a co-chaperone because of its molecular chaperone activity. Its primary role is to recognize client substrates and deliver them to the downstream effector HSP70. Together, HSP40 and HSP70 form a tightly coordinated functional network [89], which may offer greater translational value than strategies directed at a single target. Furthermore, mitochondrial homeostasis is critical for determining the osteogenic differentiation of BMSCs [92]. HSP40 family members, particularly DNAJA3, contribute to the maintenance of mitochondrial membrane potential, preservation of mitochondrial DNA integrity, and regulation of apoptosis and cellular senescence. These properties suggest that HSP40 proteins may serve as key molecular hubs linking mitochondrial dysfunction to impaired bone formation. Accordingly, they may represent promising candidate biomarkers and therapeutic targets for bone metabolic disorders.

### HSPA (HSP70) family and bone metabolism

#### Overview of the HSP70 family

HSP70 proteins, as central members of the HSP family, contain two major structural domains: an N-terminal ATP-binding domain that regulates their activity, and a C-terminal domain responsible for substrate binding [93]. HSP70 proteins exhibit immunomodulatory properties. Their active peptide fragments can effectively activate natural killer (NK) cells, making them potential candidates for immunotherapy. Moreover, when used as vaccine carriers, HSP70 molecules significantly enhance antigen presentation efficiency and strengthen T-cell–mediated immune responses [94]. In addition, HSP70 plays pivotal roles in protein transport, folding, degradation, and the regulation of apoptosis [94]. Under physiological conditions, HSP70 is expressed at low basal levels; however, upon exposure to stress stimuli, its expression rapidly increases within minutes. This prompt response enables HSP70 to function as a cytoprotective factor, particularly during nuclear translocation events [95].

#### HSP70 and bone metabolism

Although HSP70 has been primarily studied as a regulator of cellular stress and apoptosis, its potential role in bone metabolism has gained increasing attention. HSP70 alleviates oxidative stress–induced mitochondrial dysfunction by suppressing the JNK/c-Jun signaling pathway, thereby protecting nucleus pulposus-derived stem cells from apoptosis and senescence, suggesting that it may play a key role in maintaining skeletal cellular homeostasis [96]. Studies have shown that mild heat stress upregulates HSP70 expression in BMSCs, which positively correlates with enhanced ALP activity and increased mineralized nodule formation. However, its expression is not affected by active vitamin D₃, suggesting that HSP70 may function independently of classical osteogenic signaling pathways [97]. Through its molecular chaperone activity, HSP70 maintains the synthesis and folding homeostasis of key osteogenic proteins such as Runx2 and Osterix. Downregulation of HSP70 results in decreased ALP activity, calcium deposition, and osteogenic transcription factor expression, thereby impairing the osteogenic potential of hBMSCs and abolishing the pro-differentiation effects of mild heat stimulation. Mechanistically, HSP70 may regulate Osterix expression through modulation of the Bone Morphogenetic Protein 2 (BMP2)/p38/ERK1/2 signaling axis. Loss of HSP70 induces protein aggregation stress, which indirectly inhibits the osteogenic differentiation process [98].

Beyond its canonical chaperone role, HSP70 also participates directly in transcriptional regulation and osteochondral lineage specification. HSP70 interacts with the conserved central domain of the transcription factor *Sox9* via its C-terminal region to form a “DNA-binding” ternary complex, which enhances *Sox9* mediated transcriptional activation of chondrogenic marker genes such as *Col2a1*. This interaction is independent of ATP/ADP hydrolysis, suggesting that HSP70 acts as a molecular scaffold rather than a classical chaperone, facilitating the assembly of the SOX9-centered osteogenic signaling network [99].

Moreover, low-intensity pulsed ultrasound (LIPUS) selectively upregulates HSP70 expression, thereby enhancing the osteogenic differentiation potential of adipose-derived stem cells (ADSCs). Mechanistically, HSP70 stabilizes the active conformation of downstream BMP effectors Smad1/5 and inhibits their ubiquitin-dependent degradation, amplifying BMP pathway–mediated transcription of osteogenic genes. This establishes a direct coupling between the inducible level of HSP70 and the rate of bone formation, underscoring its pivotal role as a mechanoresponsive regulator of bone remodeling [100].

Nevertheless, HSPA5, a member of the HSP70 family, negatively regulates the osteogenic differentiation of BMSCs while promoting their adipogenic differentiation, thereby disrupting the balance between bone formation and adipogenesis and contributing to the pathogenesis of OP. Elevated expression of HSPA5 suppresses the activation of the PERK–eIF2α–ATF4 signaling pathway, thus inhibiting the transcription of osteogenesis-related genes. In contrast, treatment with the HSPA5 inhibitor HA15 induces moderate ER stress, activates autophagy, and reduces osteoblast apoptosis, ultimately restoring the dominance of bone formation [101]. During bone repair, although the migration of BMSCs to injury sites is a critical step, alterations in HSP70 expression appear to have a limited effect on their migratory capacity, suggesting that HSP70 is not a key regulator of BMSCs migration [102].

OBs are essential cells in bone development and repair, forming new bone tissue by secreting extracellular matrix (ECM) components and promoting their subsequent mineralization. As a molecular chaperone, HSP70 maintains osteoblast viability by suppressing oxidative stress and apoptosis pathways, including the Bax/caspase-3 cascade. Mild heat stimulation combined with cyclic stretching upregulates HSP70 expression by approximately 5.5-fold in osteoblastic precursor MC3T3–E1 cells, while concurrently enhancing the transcription of osteogenic markers osteopontin (OPN) and osteoprotegerin (OPG) and inhibiting the expression of the osteoclast-related protein MMP-9 [103].

HSP70 negatively regulates PGE1-induced IL-6 synthesis in osteoblast precursor cells MC3T3–E1 through its molecular chaperone function, mechanism-wise inhibiting p38 MAPK phosphorylation, thereby suppressing the expression of downstream inflammatory factors [104]. The mitochondrial isoform of HSP70, mt-HSP70 (HSPA9), plays a non-classical role in bone metabolism regulation. When Col I misfolds in OBs, its expression is upregulated and participates in activating the integrated stress response (ISR). mt-HSP70 may sense structural perturbations at the ER-mitochondria contact sites, collaborating with the transcription factor ATF5 to regulate ISR activity, thereby maintaining normal osteoblastic mineralization [105].

In some cases, HSP70 exhibits a “Janus-faced” effect in bone metabolism, with its net effect depending on intracellular and extracellular localization and the status of the immune microenvironment. HSP70 inhibits HLA-B27 misfolding, alleviates ER stress, and improves antigen presentation, indirectly suppressing inflammation-driven upregulation of RANKL expression, thereby blocking OCs differentiation and activation, exerting a bone-protective effect. However, eHSP70, as a danger signal, can induce macrophages to secrete TNF-α, IL-1β, and IL-6 through a CD14-dependent pathway. These cytokines enhance RANKL expression by activating the NF-κB pathway and suppress the Runx2/Wnt signaling in osteoblasts, ultimately exacerbating bone resorption [106].

#### Potential clinical value of the HSP70 family in osteoporosis

HSP70 isoforms may exert distinct, and in some cases opposing, biological effects during the development and progression of OP. Therefore, HSP70 should not be viewed clinically as a single homogeneous target. Instead, isoform-specific differences should be considered to improve the precision of biomarker discovery and therapeutic strategy development. In an ovariectomized mouse model of OP, reduced HSPA1 expression in bone marrow B cells promoted OC formation and bone resorption [107], suggesting that HSPA1 deficiency may be associated with an OP phenotype characterized predominantly by increased osteoclastic activity. In contrast, HSPA5, a major regulator of endoplasmic reticulum stress within the HSP70 family, was markedly upregulated in OBs in femoral sections from OP mice and negatively regulated the osteogenic differentiation of BMSCs [101]. This finding suggests that HSPA5 may be more closely involved in pathological processes characterized primarily by impaired osteogenesis. Another HSP70 isoform, HSPA8, has also been implicated in osteoclast regulation. In situ experiments showed that HSPA8 knockdown significantly inhibited RANKL-induced OC formation [108]. Together, these findings demonstrate that individual HSP70 isoforms play non-uniform roles in bone remodeling imbalance.

The biological effects of HSP70 are also strongly influenced by subcellular localization. Intracellular HSP70 promotes bone formation [103], whereas eHSP70 stimulates inflammation and enhances bone resorption [106]. This distinction indicates that clinical studies should differentiate between intracellular and extracellular HSP70, as well as the corresponding sample sources, to more accurately define their roles across disease stages and bone remodeling states.

In both in vitro and in vivo studies, physical interventions such as moderate heat and mechanical stimulation promoted bone formation by inducing HSP70 expression [97, 98, 100]. These findings suggest that rehabilitation strategies based on such stimuli may contribute to restoration of the bone microenvironment. In addition, HSP70 occupies a central position within the HSP cooperative network and interacts functionally with both HSP40 and HSP110 [89, 109]. This network may therefore provide an important molecular basis for the bone-reparative effects of integrated rehabilitation approaches, including thermotherapy and mechanical stimulation.

mt-HSP70 is particularly noteworthy. It not only protects the mineralization capacity of osteoblasts, but may also represent a key translational node linking endoplasmic reticulum stress, mitochondrial dysfunction, and impaired bone formation [105]. This observation suggests that, beyond conventional anabolic and anti-resorptive therapies, targeting mitochondria-endoplasmic reticulum crosstalk may offer a novel strategy for improving osteogenic function. However, its clinical relevance remains to be confirmed in large-scale population studies.

### HSPC (HSP90) family and bone metabolism

#### Overview of HSP90 family

The *Hsp90* encoding gene in the human genome is located at 14q32.33, and it is a highly conserved ATP-dependent molecular chaperone. Its functional structure can be divided into three major modules: the N-terminal domain (NTD), the middle domain (MD), and the C-terminal domain (CTD). The NTD contains the ATP binding site, which is the primary target for most HSP90 inhibitors; the MD is involved in client protein recognition and binding; and the CTD primarily mediates protein dimerization and interactions with co-chaperones [110]. HSP90 exists in various isoforms in higher eukaryotes, including cytosolic HSP90α, HSP90β, ER HSP90 (GRP94/HSP90B1), and mitochondrial HSP90 (TRAP1) [110]. As a molecular chaperone, HSP90 interacts with various client proteins to regulate cellular adaptation to stress, participate in the maintenance and enhancement of growth signaling pathways, and indirectly influence cell death mechanisms [111].

#### HSP90 and bone metabolism

Recent studies have found that HSP90, in addition to its role as a molecular chaperone involved in protein folding and stabilization, also plays a crucial role in regulating bone metabolism. Bisphosphonates can induce OBs to release ATP, activating the P2Y receptor/ERK/HSP90 signaling axis to promote the proliferation of osteoblast-like cell lines (such as HOBIT and MG-63), with HSP90 expression upregulation dependent on ERK activation and transcriptional processes [112]. Mechanical stimuli, such as low-intensity pulsed ultrasound, can upregulate HSP90, which, even when the classic bone-related signaling pathway BMP is inhibited, still maintains Smad1 phosphorylation, enhancing the viability and mineralization of OBs derived from mouse calvaria. This suggests that HSP90 plays a key role in a non-classical osteogenic signaling pathway [113]. HSP90 is also a key molecule coupling bone formation signaling with OB localization. In OB precursor cell line MC3T3–E1, HSP90 positively regulates PDGF-BB-induced migration by maintaining the phosphorylation levels of p44/p42 MAPK, and pharmacological inhibition of HSP90 significantly weakens this process [114]. However, some studies show that cytosolic HSP90 may negatively regulate bone remodeling. In OB precursor cell line MC3T3–E1, it continuously inhibits PGF2α-induced IL-6 transcription and secretion in a p38 MAPK-dependent manner, and pharmacological inhibition or siRNA knockdown of HSP90 can relieve this inhibitory effect [115].

HSP90α/β directly regulates osteogenic fate by stabilizing the glucocorticoid receptor (GR). After blocking its chaperone function with the HSP90 inhibitor 17-AAG, GR nuclear translocation is suppressed in C3H10T1/2 and primary OBs, relieving the transcriptional inhibition of ALP/Col I by high-dose dexamethasone, restoring mineralized nodule formation and surpassing bone resorption. Ultimately, this reverses glucocorticoid-induced OP in C57BL/6 and BALB/c mice [116].

HSP90α is the major inducible isoform of HSP90, typically upregulated under cellular stress conditions [110]. Under stress conditions such as hypoxia and serum deprivation, HSP90α inhibits BMSCs apoptosis and enhances NO production through the PI3K-Akt and ERK1/2 pathways, thereby maintaining the quantity, function, and osteogenic potential of BMSCs. Its inhibitors can reverse this effect, suggesting that HSP90α is a potential regulatory node in bone metabolism homeostasis [117]. Directed migration of BMSCs is a crucial step in bone remodeling. HSP90α upregulates CXCR4 expression through the PI3K-Akt and ERK1/2 pathways, promoting BMSC migration to bone defect sites, and working synergistically with the bone matrix barrier function to recruit osteoprogenitor cells and subsequent osteogenic differentiation in the defect area [118]. HSP90β is a constitutively expressed isoform of HSP90, maintaining basal protein stability and folding function under normal physiological conditions [110]. HSP90β, highly expressed in human umbilical mesenchymal stem cells, inhibits the dissociation of the cyclin D1/E-cdk2 complex, extending G1/S phase arrest, thereby limiting Runx2-mediated osteogenic differentiation. Targeted knockdown of HSP90β can relieve this inhibition, significantly increasing osteocalcin expression and mineralized nodule formation [119].

In OCs, HSP90 inhibits the activation of the non-receptor tyrosine kinase c-Src through its co-chaperone protein Cdc37, thereby promoting OCs apoptosis and limiting bone resorption. 5-azacytidine-induced protein 2 binds to the HSP90-Cdc37 complex, enhancing HSP90‘s negative regulation of c-Src [120]. HSP90α/β interacts with Rab11b through its ATPase domain, inhibiting bone resorption in the RANKL-induced RAW-D OC differentiation model. Pharmacological inhibition of HSP90 with 17-AAG or siRNA knockdown of its isoforms can relieve this inhibition, significantly promoting OCs formation and function [121].

In addition to the direct inhibitory effect of HSP90 on OCs, several HSP90 inhibitors (which suppress HSP90 expression) have been found to enhance OC differentiation and viability, increasing the risk of bone metastasis during cancer treatment. After the ATP binding sites of HSP90α/β isoforms are occupied by inhibitors such as 17-AAG, RANKL-M-CSF-induced OC differentiation and fusion are enhanced, leading to a significant increase in OC numbers on the bone surface and a decrease in trabecular bone mass [122]. HSP90 inhibitors (such as 17-AAG, CCT018159, and NVP-AUY922) significantly enhance RANKL-induced OC differentiation in the RAW264.7 macrophage cell line and bone marrow-derived macrophages. This mechanism is independent of classical transcription factors such as NFATc1, NF-κB, and c-Fos, and instead involves upregulation of MITF expression and activation of its target gene *VATPase-d2* promoter activity [123].

HSP90 plays an inhibitory role in CC differentiation and growth plate vascularization. The selective inhibitor 17-DMAG upregulates VEGF receptor Flk-1, restores HIF-1α stability, and reactivates growth plate vascularization while correcting CC differentiation defects in the tibial cartilage dysplasia chicken model [124]. Similarly, the natural inhibitor Tripterygium wilfordii significantly downregulates HSP90 expression in growth plate CCs and upregulates its VEGF receptor Flk-1, thereby restoring angiogenesis, promoting cartilage calcification, and correcting tibial cartilage dysplasia [125]. Hypoxia is considered an important regulatory factor in cartilage development, and HIF-1α is the major regulatory factor in the hypoxic response. In tibial dyschondroplasia lesions, the hypoxia-HIF-1α-HSP70/90 pathway is activated, and upregulated HSP90 influences CCs survival, differentiation, and apoptosis [126]. However, some studies suggest that the net benefit of HSP90 is favorable to cartilage synthesis. It is a key node in regulating the balance between anabolism and catabolism. Loss of its activity can simultaneously block IGF-1-induced Akt/p42-44 phosphorylation-COL2A1 axis (anabolic signal) and inhibit IL-1β-driven MMP13 upregulation (catabolic signal), suggesting that HSP90 inhibition may preferentially weaken catabolic reactions, providing a theoretical basis for precise intervention in osteoarthritis [127].

#### Potential clinical value of the HSP90 family in osteoporosis

The potential clinical value of the HSP90 family in OP mainly arises from its isoform-specific roles and functional pleiotropy in the regulation of bone metabolism. Current evidence indicates that different HSP90 family members exert inconsistent, and in some cases opposing, biological effects during osteogenesis and osteoclastogenesis. HSP90α can, to some extent, maintain the survival, migration, and osteogenic potential of BMSCs [117], yet it may also promote osteoclast differentiation [37]. HSP90β similarly exhibits bidirectional regulatory features: on the one hand, it can enhance BMP-2-mediated expression of osteogenesis-related genes [128]; on the other hand, it may inhibit the osteogenic differentiation of BMSCs [119]. These findings suggest that the HSP90 family does not function simply as a pro-osteogenic or anti-osteogenic factor, but rather may serve as an important molecular hub for maintaining the dynamic balance of bone remodeling. Accordingly, abnormal expression and functional dysregulation of HSP90 family members may reflect distinct states of bone metabolic imbalance and provide a basis for the molecular subtyping of OP. Notably, the endoplasmic reticulum-localized member GRP94 shows significantly altered expression in patients with osteoporosis and demonstrates a certain degree of diagnostic performance, suggesting that it may serve as a candidate biomarker with translational potential [129]. Meanwhile, the mitochondrial member TRAP1 may contribute to the maintenance of bone homeostasis through regulation of energy metabolism. Although its precise mechanism remains to be clarified, the emergence of selective regulatory strategies has opened new directions for future targeted development [130].

Although no artificial intelligence-based predictive model specifically targeting HSP90 in OP has yet been established, existing studies have begun to demonstrate its potential applicability. In the therapeutic field, researchers have preliminarily used machine learning to predict the efficacy of the HSP90 inhibitor 17-AAG in prostate cancer [131]. In contrast, conventional osteoporosis risk assessment tools, such as FRAX, do not incorporate biomarkers, whereas modern machine-learning approaches can integrate multisource data to identify complex patterns and improve predictive accuracy [132]. Therefore, future integration of HSP90-related biomarkers with machine-learning methods may offer new opportunities for early identification, risk stratification, and individualized intervention in OP. It should be noted, however, that the HSP90 family is broadly involved in the maintenance of cellular homeostasis and physiological processes across multiple tissues, and systemic intervention may therefore induce adverse effects outside the skeletal system. In addition, the specific roles of different isoforms and their cell type-dependent effects have not yet been fully elucidated, which further limits their direct clinical application.

### HSPH (HSP110) family and bone metabolism

#### Overview of the HSP110 family

The HSP110 protein is encoded by the *Hsph1* gene located on the short arm of chromosome 13 at region 12.3, which contains 20 exons. As a member of the HSP70 superfamily, HSP110 primarily acts as a nucleotide exchange factor, working in concert with HSP70 to regulate protein folding [133]. When HSP70 binds to misfolded proteins, ATP is hydrolyzed to ADP, leading HSP70 into a stable ADP-bound state. Subsequently, HSP110 interacts with the nucleotide-binding domain of HSP70, promoting ADP release and restoring the ATP-bound state of HSP70, thereby reactivating its chaperone activity [109].

## HSP110 and bone metabolism

HSP110 is consistently highly expressed in the proliferative and hypertrophic zones of CCs in the rat tibial growth plate, with its spatial distribution partially overlapping with the apoptotic initiation zones detected by TUNEL. This suggests that the molecule may directly participate in regulating the apoptosis process of growth plate CCs, thereby coupling cellular clearance and matrix calcification in cartilage ossification. Immunohistochemical analysis shows that HSP110 signaling extends from the proliferative zone to the upper part of the hypertrophic zone, whereas HSP28/HSP70 are confined to the upper part of the hypertrophic zone. This indicates that HSP110 has an earlier regulatory window in the CC differentiation-apoptosis-calcification sequence [134].

### Potential clinical value of the HSP110 family in osteoporosis

Direct evidence linking HSP110 to the pathogenesis of OP remains limited. Its best-characterized function is as a nucleotide exchange factor (NEF) for HSP70, through which it cooperates with HSP70 to regulate protein folding [133]. In addition, a small number of studies suggest that HSP110 may be involved in OP-related cellular stress responses, particularly through associations with apoptosis and calcification during endochondral ossification [134]. Overall, the potential clinical application of HSP110 in OP currently rests primarily on its functional cooperation with other heat shock proteins.

To provide a systematic overview of the mechanistic basis underlying HSP-mediated regulation of bone metabolism, the major HSP family members, their target cells, signaling pathways, and functional directions are summarized in Table 1.

**Table 1:** Roles, signaling mechanisms, and functional directions of HSP family members in bone metabolism

HSP Family / Member	Subcellular Localization	Major Target Cells	Upstream Stimuli	Key Signaling Pathways / Molecular Mechanisms	Functional Direction (↑Promoting / ↓Inhibiting)	Biological Function Summary	Experimental Evidence	Major Findings	Representative References
HSPB1 (HSP27)	Cytoplasm, cytoskeleton-associated	OBs, BMSCs, OCs	Heat, electromagnetic stimulation, ROS	p38 MAPK / Akt / NF-κB inhibition, ROS clearance, phosphorylation at Ser15/78/82	↑ Osteogenesis ↓Osteoclastogenesis	Promotes osteoblast differentiation, stabilizes cytoskeleton, reduces ROS-mediated apoptosis; suppresses osteoclast differentiation	In vivo (mouse bone defect), in vitro (BMSCs osteogenic induction)	HSPB1 upregulation activates p38 MAPK, enhances ALP expression, and inhibits RANKL-induced osteoclast formation	[33, 56, 59, 60, 62]
HSPB5 (CRYAB, αB-crystallin)	Cytoplasm, cytoskeleton, nucleus	BMSCs, CCs	Inflammation, oxidative stress, bone defect	Wnt/β-catenin stabilization, FTH1–ferroptosis inhibition, caspase-3/Bax anti-apoptotic regulation	↑ Osteogenesis ↑ Anti-apoptosis	Stabilizes β-catenin, protects against oxidative stress, inhibits apoptosis, enhances osteogenic differentiation of BMSCs	In vitro, in vivo (osteoporotic model)	CRYAB overexpression enhances osteogenic gene expression and reduces bone loss	[68, 70, 71, 73]
HSPB7	Cytoplasm	BMSCs	Osteogenic induction	ERK1/2 phosphorylation regulation	↓ Osteogenesis	Downregulated during osteogenic differentiation; knockdown activates ERK and promotes ALP activity	In vitro	HSPB7 acts as a negative regulator of osteogenesis	[75, 76]
HSP40 (DNAJ)	Cytoplasm	BMSCs, OBs	Luteolin induction, oxidative stress	HSP40–HSP70 chaperone complex; PPARγ inhibition / RUNX2 activation	↑ Osteogenesis ↓ Adipogenesis	Promotes RUNX2 nuclear translocation and suppresses adipogenic differentiation	In vitro	Luteolin upregulates DNAJ, enhancing osteogenic gene transcription	[90, 91]
HSP70 (HSPA1A, HSPA9)	Cytoplasm, mitochondria	OBs, BMSCs, CCs	Heat stress, mechanical loading, LIPUS, PEMF	BMP2–Smad, ERK, JNK inhibition, SOX9–COL2A1 activation, Osterix stabilization	↑ Osteogenesis ↑ Chondrogenesis ↓ Inflammation	Enhances mineralization, suppresses inflammation and oxidative stress, maintains mitochondrial function	In vivo (rat thermal model), in vitro (OB/BMSC cultures)	LIPUS upregulates HSP70, activating BMP2–Smad signaling to promote bone formation	[97–100]
HSPA5 (GRP78)	Endoplasmic reticulum lumen	BMSCs, OBs	ER stress (PERK activation)	PERK–eIF2α–ATF4 axis	↓ Osteogenesis ↑ Adipogenesis	Excessive ER stress upregulates GRP78, inhibiting osteogenic gene transcription	In vitro	GRP78 overexpression suppresses Runx2; HA15 inhibitor restores osteogenesis	[101]
HSP90α/β	Cytoplasm	OBs, OCs, BMSCs	Hormonal or mechanical stress	ERK, PI3K–Akt, glucocorticoid receptor (GR) complex	Dual: ↑ or ↓ depending on context	Promotes OB migration but can also enhance OC resorption under stress	In vivo (glucocorticoid-induced OP model), in vitro	HSP90 inhibitor 17-AAG reverses glucocorticoid-induced bone loss; excessive inhibition may stimulate OC activity	[116, 118, 122]
HSP90B1 (GRP94)	Endoplasmic reticulum	OBs, CCs	Hypoxia, ER stress	VEGF–Flk1 / HIF-1α stabilization	↑ Angiogenesis ↑ Chondrogenesis	Assists VEGF folding, promotes vascularization and cartilage formation	In vivo / in vitro	GRP94 inhibition restores angiogenic function and bone development	[124, 125, 129]
TRAP1 (mitochondrial HSP90)	Mitochondrial matrix	OBs	Metabolic stress	ATPase modulation, mitochondrial protection	↑ Metabolic homeostasis	Maintains mitochondrial bioenergetic balance and anti-apoptotic capacity	In vitro	TRAP1 ATPase regulation protects osteoblast metabolism	[130]
HSP110 (HSPH1)	Cytoplasm	CCs	Bone development	Nucleotide exchange factor (NEF) for HSP70	↑ Chondrocyte hypertrophy and mineralization	Regulates chondrocyte apoptosis–mineralization coupling	Histological analysis	HSP110 participates in growth plate chondrocyte maturation and mineral coupling	[134]

**Symbols**:↑indicates promoting/upregulating effect;↓indicates inhibiting/downregulating effect. **References**: Numbers in brackets correspond to reference list entries. **Functional direction**: Slash-separated entries demonstrate context-dependent dual roles. **Experimental evidence**: “In vivo” and “in vitro” denote animal models and cell culture studies, respectively. HSP, heat shock protein; OBs, osteoblasts; BMSCs, bone marrow-derived mesenchymal stem cells; OCs, osteoclasts; ROS, reactive oxygen species; MAPK, mitogen-activated protein kinase; NF-κB, nuclear factor kappa-light-chain-enhancer of activated B cells; ALP, alkaline phosphatase; RANKL, receptor activator of nuclear factor kappa-Β ligand; ERK, extracellular signal-regulated kinase; SOX9, SRY-box transcription factor 9; COL2A1, collagen type II alpha 1 chain; BMP2, bone morphogenetic protein 2; PPARγ, peroxisome proliferator-activated receptor gamma; RUNX2, runt-related transcription factor 2; GRP78, glucose-regulated protein 78; HIF-1α, hypoxia-inducible factor-1 alpha; VEGF, vascular endothelial growth factor; FTH1, ferritin heavy chain 1

On the basis of these mechanistic insights, the potential translational relevance of different HSP family members in OP is further summarized in Table 2, with emphasis on their possible roles in clinical stratification, biomarker development, and therapeutic targeting.

**Table 2:** Potential translational value of heat shock protein family members in osteoporosis

HSP family/member	Core role in bone metabolism	Clinical relevance	Translational positioning	Potential clinical applications	Translational readiness/key limitations	Representative References
HSPB (HSPB1 / HSPB5 / HSPB7)	HSPB1: Pro-osteogenic and anti-osteoclastogenic, helping maintain bone remodeling balance.HSPB5: Stabilizes β-catenin and exerts anti-apoptotic/anti-ferroptotic effects to promote osteogenesis. HSPB7: A negative regulator of osteogenesis that suppresses osteogenic differentiation and mineralization.	HSPB5: Decreased in the plasma of patients with OP, suggesting an impaired osteogenesis/high marrow adiposity subtype. HSPB7: Increased in BMSCs from OP models, suggesting intrinsic suppression of osteogenesis.	Biomarker-based stratification; preconditioning target for cell therapy; candidate for bone-targeted protein/nucleic acid delivery.	Identification of patients more suitable for osteoanabolic combination therapy rather than anti-resorptive monotherapy; thermal preconditioning of transplanted MSCs and functionalization of biomaterials for local bone defect repair; local delivery of HSPB7 inhibition to reduce cardiac off-target risk.	Early-to-intermediate: Human biomarker signals and substantial in vitro/animal evidence are available, but unified detection thresholds, bone-targeted delivery, and prospective stratification studies are still lacking.	[33, 56–58] [69–71, 74] [76–86]
HSP40 (DNAJ / DNAJA3)	As an HSP70 co-chaperone, HSP40 acts upstream of BMSC fate determination; it promotes osteogenesis by suppressing PPARγ and enhancing RUNX2 transcriptional activity, and may link mitochondrial homeostasis to osteogenic capacity.	Direct clinical evidence is limited, but its upstream regulatory role suggests greater value as an early abnormality marker rather than an end-stage damage indicator.	Candidate early-screening biomarker; target within the HSP40–HSP70 cooperative network; mechanistic stratification node for metabolism-/mitochondrial stress-related OP.	Suitable for inclusion in multi-biomarker panels to identify patients with adipogenic shift of BMSCs and impaired mitochondrial homeostasis; may also serve as a pharmacodynamic monitoring node for natural small-molecule interventions, such as luteolin-like strategies.	Early exploratory: Evidence is mainly limited to cell and mechanistic studies, with no animal-to-patient closed-loop validation; currently more suitable as a research-oriented translational biomarker.	[89, 91, 92]
HSP70 (HSPA1A / HSPA5 / HSPA8 / HSPA9)	Different isoforms and localizations have distinct effects: intracellular HSP70 is generally pro-osteogenic and anti-inflammatory, while maintaining mitochondrial and protein homeostasis; HSPA5 tends to inhibit osteogenesis and promote adipogenesis; extracellular HSP70 may enhance inflammation and bone resorption.	Reduced HSPA1 suggests an osteoclast-active phenotype, whereas increased HSPA5 indicates impaired osteogenesis and endoplasmic reticulum stress; HSP70 should not be treated as a single marker because isoforms and intracellular or extracellular sources differ in biological significance.	Molecular stratification marker; predictor of response to rehabilitation or physical therapy; intervention node in endoplasmic reticulum–mitochondrial crosstalk; target in the extracellular HSP70 inflammatory amplification loop.	Distinguishes osteoclast driven and osteogenesis impaired patients; helps identify individuals more likely to benefit from endogenous HSP70 enhancing strategies, including low intensity pulsed ultrasound, thermal stimulation, and mechanical stimulation; high HSPA5 expression may support combination with endoplasmic reticulum stress correcting strategies.	Intermediate: Mechanistic and animal evidence is substantial, with a translational interface for rehabilitation; limitations include strong isoform heterogeneity and the need for standardized detection criteria.	[89, 97, 98] [100, 101] [103] [105–109]
HSP90 (HSP90α / HSP90β / GRP94 / TRAP1)	Isoform specific and bidirectional: affects BMSC survival, migration, and osteogenic signaling, and also participates in osteoclast differentiation, glucocorticoid receptor mediated bone loss, and endoplasmic reticulum and mitochondrial homeostasis.	Better suited as a stratification marker of imbalanced bone remodeling than as a simple high or low indicator of protection or injury; GRP94 shows differential expression and some diagnostic potential.	Target for drug repurposing and isoform selective inhibition; candidate biomarker for AI based risk stratification; companion diagnostic node for glucocorticoid related osteoporosis.	Helps identify patients likely to benefit from HSP90 and GR axis targeted intervention; future strategies may favor isoform selective inhibition or allosteric modulation rather than broad inhibition to reduce pro osteoclastogenic effects and extra skeletal toxicity.	Intermediate to frontier: Mature experience from anticancer drug development is available, but osteoporosis applications still need to address isoform selectivity, safety, and extra skeletal adverse effects.	[36, 117, 119] [128–132]
HSP110 (HSPH1)	As a nucleotide exchange factor for HSP70, it mainly participates in chondrocyte differentiation, apoptosis–mineralization coupling, and stress adaptation by enhancing HSP70 chaperone efficiency.	Direct evidence in OP is the most limited, and it is therefore unlikely to serve as a stable standalone clinical biomarker at present; it is better considered an auxiliary node in the HSP70 cooperative network.	Network level auxiliary target; can be incorporated into multi omics and AI models to improve interpretation of the heat shock response network.	Not currently suitable for standalone diagnostic or therapeutic development; better used as a combinatorial analytical factor in HSP70 related pathway studies, osteochondral interface abnormalities, and growth plate or mineralization coupling.	Proof of concept stage: Evidence remains limited to basic research, and clinical translation will likely depend on integrated modeling with the HSP70 network.	[133, 134]

**References**: Numbers in brackets correspond to reference list entries. **Clinical relevance:** Indicates the potential association of each HSP family/member with specific osteoporosis-related phenotypes or pathological states. **Translational positioning:** Refers to the possible role of each HSP family/member in biomarker development, molecular stratification, therapeutic targeting, or translational research frameworks. **Potential clinical applications:** Summarizes possible uses in patient subgrouping, prognosis assessment, intervention selection, and targeted therapeutic development. **Translational readiness/key limitations:** Reflects the current stage of evidence and the major barriers to clinical implementation, including insufficient human validation, lack of standardized thresholds, limited bone-targeted delivery, and safety concerns. HSP, heat shock protein; OP, osteoporosis; BMSCs, bone marrow-derived mesenchymal stem cells; MSCs, mesenchymal stem cells; ER, endoplasmic reticulum; AI, artificial intelligence; GR, glucocorticoid receptor

#### HSPs as therapeutic targets for osteoporosis: translational medical prospects

##### The synergistic regulatory network of HSPs in the bone immune microenvironment–“from molecular chaperones to immune sentinels”

**Fig. 2:**
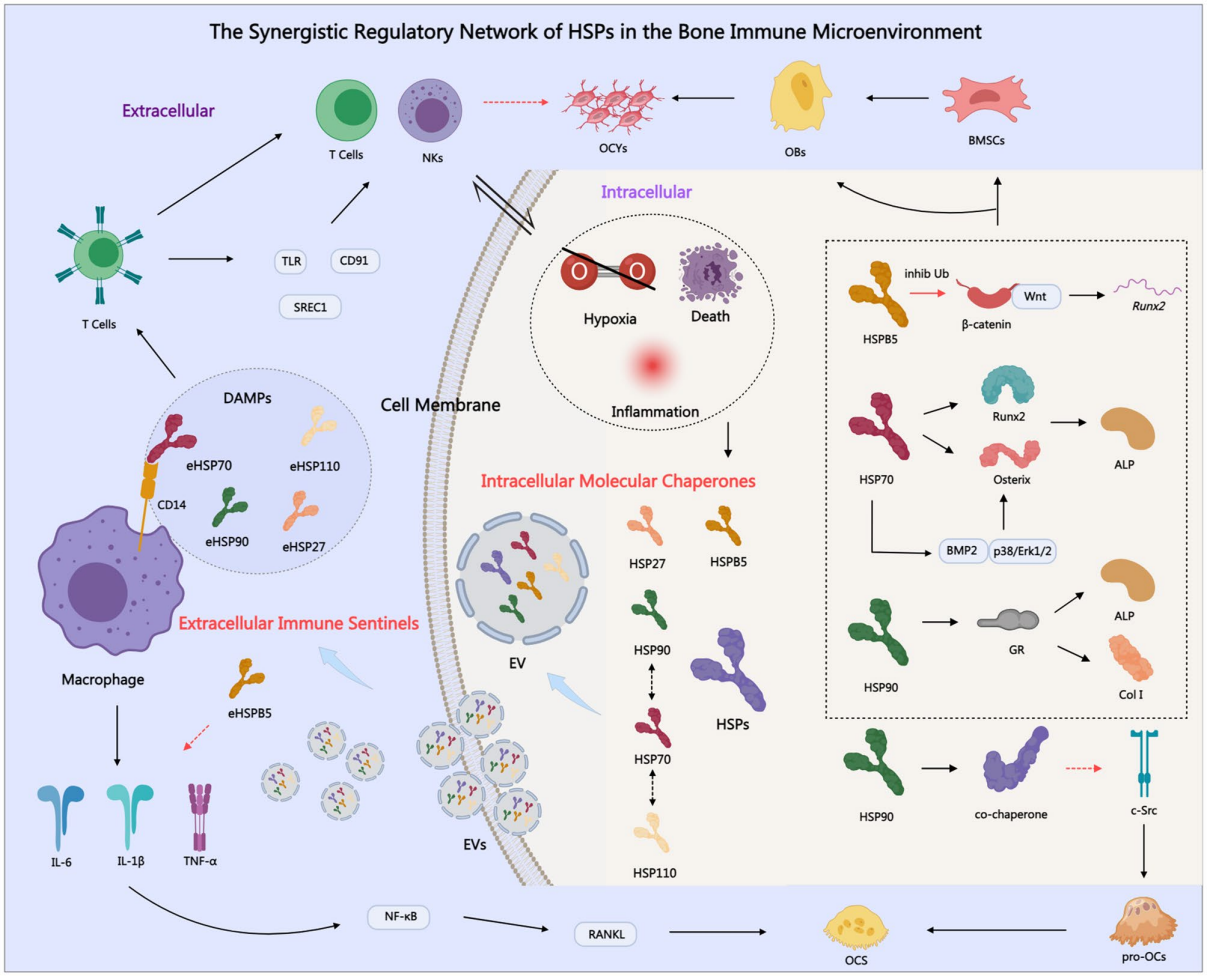
The spatiotemporal regulatory network of HSPs as “intracellular chaperones” and “extracellular sentinels” in the bone immune microenvironment. This figure depicts the precise network of HSPs in the regulation of bone immunity. Within the cell, HSPs (such as HSP70, HSP27, and HSP90) function as molecular chaperones, modulating key signaling pathways, including p38 MAPK, c-Src, and NF-κB, to influence osteogenic differentiation processes mediated by the Wnt/β-catenin, Runx2, and Osterix pathways. These proteins also contribute to the maintenance of intracellular homeostasis and cell survival. In response to external stimuli, such as hypoxia or inflammation, intracellular HSPs can be transferred to the extracellular environment via mechanisms like extracellular vesicles or exosomes. Once in the extracellular space, HSPs (e.g., eHSP70, eHSP90, eHSP27, eHSP110) act as “immune sentinels” and DAMPs. They interact with surface receptors on immune cells, including macrophages and T cells (e.g., TLR, CD91, SREC1), thereby regulating the release of pro-inflammatory cytokines, such as IL-6, IL-1β, and TNF-α, and enhancing the immune surveillance function of NK cells. Furthermore, extracellular HSPs are involved in the modulation of the RANKL/NF-κB signaling pathway, which influences the differentiation and activation of OCs. ALP: alkaline phosphatase; BMP2: Bone Morphogenetic Protein 2; BMSCs: bone marrow mesenchymal stem cells; Col I: Collagen I; C-SRC: Cellular Src tyrosine kinase; DAMPs: damage-associated molecular patterns; ERK: Extracellular Signal-Regulated Kinase; EVs: Extracellular Vesicles; GR: glucocorticoid receptor; BMSCs: bone marrow mesenchymal stem cells; HSPs: Heat shock proteins; OBs: osteoblasts; OCs: osteoclasts; OCYs: Osteocytes; OP: Osteoporosis; RANKL: Receptor Activator of Nuclear Factor-κB Ligand; Runx2: Runt-related transcription factor 2; SREC1: Sialic acid-binding immunoglobulin-like lectin 1; TLR: Toll-like Receptor. (the figure was created in Medpeer)

The role of HSPs in the bone immune microenvironment of OP goes far beyond that of a traditional “molecular chaperone.” As shown in Fig. 2, Their function has undergone a profound evolution: from “guardians” maintaining protein homeostasis within cells to “sentinels” transmitting danger signals between cells and orchestrating immune responses. This duality and transition of roles form the core of their regulatory network.

### Molecular chaperones in maintaining homeostasis

This is the most classic role of HSPs, where they directly regulate cell fate by maintaining protein homeostasis within various cells of the bone immune microenvironment. HSPB5, as a molecular chaperone, regulates the Wnt osteogenic signaling pathway by stabilizing β-catenin, playing a key regulatory role [70]. In BMSCs and OBs, HSP70 maintains the osteogenic function by ensuring the correct folding of key osteogenic transcription factors, such as *Runx2* and *Osterix* [98]. HSP90 influences OB metabolic responses by regulating the conformational state of client proteins, such as the GR [116], maintaining cellular homeostasis and ensuring osteogenic capacity. In OC precursor cells, HSP90 forms a complex with co-chaperone proteins, inhibiting the activation of kinases such as c-Src, intrinsically suppressing excessive differentiation and survival of OCs, thereby acting as an intrinsic brake to constrain bone resorption [120]. HSPs, as molecular chaperones, ensure normal physiological functions by maintaining protein homeostasis within individual cells, making them key regulatory factors in bone homeostasis.

### Transition from “molecular chaperones” to “immune sentinels”

Upon encountering severe stress, such as inflammation, hypoxia, or cell death, the role of HSPs undergoes a fundamental shift. They are released from the intracellular compartment, either passively or actively secreted into the extracellular space. Their function changes from managing protein homeostasis to facilitating intercellular communication. Extracellular HSP70 (eHSP70) serves as a core “immune sentinel”; once it enters the extracellular environment, it no longer functions as a chaperone but is recognized by the immune system as DAMP. By binding to pattern recognition receptors such as CD14 and TLRs, eHSP70 activates classical inflammatory pathways, including the NF-κB signaling pathway, leading to the secretion of proinflammatory cytokines such as TNF-α, IL-1β, and IL-6 from macrophages [106]. Other HSPs, such as HSP27, HSP90, and HSP110, can also be actively released in either free or vesicular forms, acting as “intercellular communicators.” These chaperones amplify danger signals through receptors like CD91, TLRs, and SREC-1, triggering the activation of antigen-presenting cells and natural killer cells. This cascade ultimately leads to the reshaping of the immune microenvironment [135].

### Construction of the cooperative Network

HSP110, acting as a nucleotide exchange factor for HSP70, enhances its chaperone efficiency and functions as an “amplifier” within the chaperone network [109]. HSP40 delivers client proteins to HSP70, jointly determining the fate of proteins, and thus functions as a “scheduler” in this network [89]. Meanwhile, HSPB5, in addition to its chaperone role, directly binds to proinflammatory proteins, acting as a “neutralizing sentinel [66]. Intracellular HSP70 works tirelessly to protect OBs from stress, promoting bone formation [103], while its extracellular form, eHSP70, activates inflammation and stimulates bone resorption [106]. The dual roles of individual HSP molecules weave a cooperative network that influences the bone immune microenvironment. This insight provides a new therapeutic perspective, highlighting the need not only to monitor HSPs expression levels but also to consider their localization and form.

### Therapeutic strategies targeting HSPs: from basic research to clinical application, and from “intervenable” to “translatable”

Given the multifaceted regulatory roles of the HSP family in the bone immune microenvironment, developing targeted therapeutic strategies aimed at specific HSP molecules has emerged as a novel direction for the intervention of OP. These strategies encompass a variety of approaches, including small molecule drugs, biologics, physical therapy, and gene or cell-based therapies.

#### Small molecule drug strategies: modulating HSPs expression and function

Small molecule agents represent a priority class of HSP-targeted therapies because they benefit from relatively mature drug-development frameworks, adjustable pharmacological properties, and the feasibility of repeated administration [136–138]. Among these agents, conventional HSP90 inhibitors are the most extensively studied and best characterized. However, most act as broad-spectrum pan-inhibitors with limited selectivity, targeting the four major HSP90 isoforms, including cytosolic HSP90α/β, endoplasmic reticulum-localized GRP94, and mitochondrial TRAP1 [139]. These compounds inhibit the ATPase activity of HSP90, thereby promoting degradation of multiple client proteins that require HSP90 for proper folding and enabling simultaneous suppression of several disease-related signaling pathways, particularly in cancer. Nevertheless, this broad mechanism is also associated with adverse effects, including heat shock responses, retinopathy, and gastrointestinal toxicity [140, 141]. HSP90-targeted small molecules have progressed beyond the preclinical and early clinical stages. Notably, pimitespib (TAS-116), an orally available N-terminal ATP-competitive inhibitor selective for cytosolic HSP90α/β, has been approved in Japan for the treatment of advanced gastrointestinal stromal tumors and has shown favorable tolerability and efficacy in real-world clinical practice [142].

Given the relatively advanced development of HSP90 inhibitors in oncology and other diseases, extending their application to bone metabolic disorders such as OP is a plausible therapeutic strategy. Studies have shown that the HSP90 inhibitor 17-AAG can block the interaction between HSP90 and the GR, inhibiting GR nuclear translocation and thereby reversing glucocorticoid-induced OP [116]. However, the advantages, limitations, and translational feasibility of different intervention approaches differ substantially. Broad-spectrum HSP90 inhibitors are supported by a more established development framework and are therefore useful for early mechanistic studies. However, because they simultaneously target multiple isoforms and client proteins, they may disturb bone metabolic homeostasis and even promote osteoclastogenesis, ultimately leading to bone loss. For example, 17-AAG has been reported to produce such adverse effects in some studies [122]. By contrast, isoform-selective inhibitors may offer greater specificity and lower toxicity. KUNB31, a selective inhibitor of cytosolic Hsp90β, has shown a more manageable safety profile than conventional broad-spectrum inhibitors [139], indicating greater translational promise. In addition, allosteric modulators offer a more refined approach to isoform-specific regulation. By targeting a distinct allosteric pocket in the mitochondrial HSP90 isoform TRAP1, these compounds selectively inhibit TRAP1 without affecting HSP90α [143]. Overall, broad-spectrum inhibition is better suited for mechanistic investigation, isoform-selective inhibition is more consistent with the requirements of clinical translation, and allosteric modulation may represent a particularly promising direction for future therapeutic optimization.

Another representative small-molecule agent is the thiol antibiotic thiolutin, which potently induces HSPB1 phosphorylation., and significantly induces HSPB1 phosphorylation via the p38 MAPK pathway. In OBs and BMSCs models, this effect is closely associated with an enhanced osteogenic phenotype, suggesting its unique value as a HSPB1 phosphorylation agonist in bone metabolism research. However, its high toxicity limits its clinical application [62]. To circumvent its side effects, researchers synthesized prodrug chelators, which can be designed to remain in a “masked state” in off-target environments and release the active thiol group only under specific enzymatic conditions or in target tissues, such as bone [144]. Because active sulfhydryl groups are highly reactive, conjugation with bone-targeting moieties may improve the therapeutic potential of these agents. Candidate targeting groups include bisphosphonates [145] and other bone-affinitive molecules, such as tetracycline derivatives [146], which may enhance drug accumulation on the hydroxyapatite surface. A major challenge for further development is to achieve sufficient drug concentration at bone sites while minimizing off-target toxicity associated with high-dose administration [147].

Natural products are considered an important source of agents that regulate the expression and function of HSPs because of their structural diversity and multitarget properties. Flavonoid natural products such as luteolin can markedly induce the expression of DNAJ, a member of the HSP40 family, thereby reducing the stability of PPARγ and enhancing RUNX2 activity. This, in turn, promotes the osteogenic differentiation of BMSCs while inhibiting their adipogenic differentiation [91]. Numerous natural flavonoids, including icariin [148], naringin [149], and kaempferol [150], have shown potential to modulate the osteoimmune microenvironment and restore bone metabolic balance. Among these compounds, genistein has the strongest clinical basis, with evidence from randomized controlled trials [151, 152] Flavonoids are commonly derived from dietary sources or traditional herbal medicines and generally have favorable safety profiles. However, many exhibit low in vivo bioavailability and limited pharmacological potency. Consequently, current research has focused on improving their translational potential through nanotechnology-based delivery systems, carrier design, computer-assisted structural optimization, and combination therapy strategies [153–155]. Polyphenolic compounds may exert biological effects broadly similar to those of flavonoids. For example, epigallocatechin gallate (EGCG) directly regulate the Akt-HSP27 pathway and modulate antioxidant mechanisms, thereby influencing the differentiation, apoptosis, and survival of bone cells, ultimately promoting bone formation and inhibiting bone resorption [156]. In ovariectomized animal models, oral administration of EGCG inhibits osteoclast formation and attenuates bone loss [157].

In a randomized controlled trial of overweight or obese postmenopausal women, long-term supplementation with decaffeinated green tea extract (containing EGCG) did not significantly reduce body fat or improve bone density [158]. This finding suggests that positive results observed in experimental models have not yet translated into clear clinical benefit. Other polyphenolic compounds, such as resveratrol, have shown modest benefits for bone mineral density and bone formation markers in small-scale studies [159]. These compounds offer several advantages, including favorable safety profiles, abundant natural sources, and suitability for long-term use. However, their biological effects are generally mild and may be substantially influenced by interindividual metabolic variation.

Triterpenoid compounds represent another promising class of small molecules because they often exhibit strong biological activity and greater target specificity. Natural triterpenoids such as celastrol can function as HSP90 inhibitors. In a cleidocranial dysplasia model, celastrol significantly reduced HSP90 expression, increased expression of the VEGF receptor Flk-1, promoted angiogenesis, and ameliorated cartilage developmental defects [125]. In addition, triterpenoid compounds can exert HSP90-inhibitory, antitumor, and anti-inflammatory effects at nanomolar concentrations, indicating potent biological activity. However, their translational application is limited by several major drawbacks, including poor water solubility, low bioavailability, substantial hepatorenal toxicity, and a narrow therapeutic window [160, 161]. Furthermore, some highly active triterpenoids are derived from rare plants or require complex extraction procedures, which restricts raw material availability and complicates large-scale industrial production [162]. As a result, the application of this class of small molecules in osteoporosis remains challenging.

#### Biologics: immunogenicity of extracellular HSPs

Due to the lack of classic secretion peptide signals, HSPs were previously considered to be intracellular molecular chaperones. However, some HSPs can enter the extracellular space via non-classical pathways (such as exosomes, microvesicles, or membrane-bound forms), and act as “signaling molecules” between cells, referred to as eHSPs [163]. eHSPs are not unidimensional proinflammatory molecules; rather, their biological effects are jointly determined by protein isoform, mode of release, concentration, receptor repertoire, and the local microenvironment. These eHSPs possess a “double-edged sword” characteristic: for example, eHSP70 can activate macrophages, promote NK cell cytotoxicity, and enhance dendritic cell cross-presentation, manifesting as immune stimulation [164]. Additionally, eHSP70 can activate macrophages via the CD14/TLR4 pathway, promoting inflammatory factor release and driving bone resorption [106], indicating that it functions as both an immune alarm signal and an amplifier of inflammation. However, under low-endotoxin preparation conditions and in specific dendritic cell/T-cell systems, HSP70 can reduce the stimulatory capacity of dendritic cells and suppress T-cell responses, suggesting that it may also participate in immune restraint [165]. Therefore, eHSP70 should be regarded as a context-dependent immunoregulatory node. Based on this characteristic of eHSP70, researchers have proposed the “stress surveillance system” theory, suggesting that damaged or stressed cells release eHSPs as “alarm” signals to activate the immune system at a distance, in order to contain the spread of damage and promote tissue repair [163].

Based on these considerations, direct neutralization of eHSP70 represents the most straightforward current strategy for biologic development. Its principal advantages are a clearly defined target and a pharmacological effect largely restricted to the extracellular compartment, which should theoretically preserve the essential intracellular chaperone functions of HSP70 to the greatest extent possible. In addition, this approach is well suited for pharmacodynamic monitoring through measurement of eHSP70 levels in serum or extracellular vesicles. In a doxorubicin-induced mouse heart failure model, administration of anti-HSP70 neutralizing antibodies significantly reduced eHSP70 levels in the myocardium and serum, leading to a decrease in neutrophils and an increase in M2 macrophages. This alleviated myocardial inflammation, fibrosis, and left ventricular dilation in the doxorubicin-treated mice, significantly improving cardiac function, and confirmed the in vivo therapeutic feasibility of targeting eHSP70 with neutralizing antibodies [166]. Current supporting evidence is derived mainly from models of injury, infection, inflammation, and cardiovascular disease rather than from direct osteoporosis models. Therefore, this approach cannot yet be considered a validated therapeutic strategy for osteoporosis [163]. For eHSP70, it acts as a “danger signal” under stress conditions to initiate repair programs. However, long-term or excessive neutralization may impair the body’s response to infection or tissue damage. A more translationally promising approach is the localized or bone-targeted delivery of neutralizing antibodies. This strategy is better aligned with the chronic, low-grade inflammatory nature of osteoporosis, as it may reduce excessive extracellular HSP signaling within the bone microenvironment while preserving the physiological functions of systemic eHSP70 in damage surveillance and tissue repair. However, its application remains limited by the lack of dedicated validation in osteoporosis-specific animal models and targeted delivery systems [167].

#### Physical therapy: non-invasive induction of endogenous HSPs Expression

Physical therapies such as low-intensity pulsed ultrasound (LIPUS) and pulsed electromagnetic fields (PEMF) have shown potential to regulate bone metabolism by inducing the expression of endogenous HSPs [168, 169]. As a noninvasive technique approved by FDA for fracture healing, LIPUS can generate micromechanical stress and mild thermal effects, thereby inducing upregulation of HSP70 and HSP90 in osteoblasts [170, 171].

Two intensities of LIPUS (20 and 30 mW/cm^2^) can significantly promote osteogenic differentiation of human adipose-derived stem cells by upregulating HSP70 and HSP90 and activating the BMP-Smad signaling pathway, demonstrating excellent in vitro bone regeneration potential [100]. A single 15-minute exposure to 30 mW/cm^2^ LIPUS can significantly enhance osteogenic mineralization capacity of mouse OBs by upregulating HSP90, inhibiting HSP27, and activating the BMP-Smad signaling pathway, maintaining osteogenic activity even when BMP signaling is blocked, suggesting an alternative osteogenic induction mechanism [113]. LIPUS has several advantages, including its noninvasive nature, favorable safety profile, established clinical use, and documented ability to promote bone repair and osteogenesis. However, LIPUS-induced HSP expression is relatively transient and therefore typically requires repeated daily stimulation. Conventional treatment protocols usually involve 20–40 minutes of daily therapy for 3–6 months, which may compromise patient adherence [168]. Pulsed electromagnetic fields rapidly alleviate pain in primary OP and promote bone formation and increase bone mineral density in secondary OP, and are widely used clinically [172]. A 1.5 mT PEMF promotes skeletal muscle cell proliferation, migration, and wound closure, inducing HSP70 and other antioxidant enzymes to attenuate oxidative inflammation and thereby accelerating tissue repair [173]. Overall, both LIPUS and PEMF can induce beneficial HSP-mediated responses through physical stress and have achieved a certain degree of clinical application. However, their therapeutic efficacy still requires further validation.

Mild heat treatment is a direct strategy for promoting bone formation because it robustly induces HSP expression through the classical heat shock response [62, 174]. In vitro studies have shown that periodic exposure of osteoblasts to heat stress at approximately 41 °C increases the expression of HSP27 and HSP70, enhances calcium deposition, and upregulates osteogenic markers [62]. Exposure of BMSCs to nonlethal 42 °C thermal stimulation transiently upregulates the anti-apoptotic heat shock proteins HSP27 and HSP70, inhibits mitochondria-mediated intrinsic apoptosis (e.g., suppresses caspase-3 activation), and improves post-transplant cell survival; however, the induced protein expression persists for only 4 days, and long-term in vivo efficacy has not been demonstrated [58]. A major limitation of this approach is the difficulty of applying heat selectively to bone tissue without affecting adjacent structures, which necessitates precise control of both temperature and exposure duration. Mild heat stress at 40–42 °C is generally beneficial, whereas temperatures above 45 °C may induce osteocyte apoptosis, cause protein damage, and ultimately impair bone tissue [174]. In addition, HSP induction is relatively transient, requiring repeated stimulation to sustain its effects. The practical feasibility of this strategy in clinical settings therefore remains uncertain.

Mechanical loading and exercise are the most physiological forms of physical stimulation. Although they are not specifically intended to induce HSP expression, they are well established as cornerstone interventions for improving bone strength [175]. Regular weight-bearing exercise subjects bone cells to cyclic stress and microdamage, thereby eliciting adaptive stress responses that include HSP induction. Studies have shown that continuous compressive stress increases the expression of HSPs, such as HSP70, in osteoblasts, thereby supporting protein folding and enhancing cell survival [176]. Mechanical loading induces HSP70 overexpression in periodontal ligament cells, wherein its chaperone function repairs denatured proteins and regulates osteogenic gene expression (e.g., *Runx2* and *Osterix*), thereby promoting cell differentiation and tissue regeneration and enhancing hard-tissue regeneration of the periodontal ligament [177]. As a foundational strategy for osteoporosis prevention, exercise is safe, cost-effective, and associated with broad systemic health benefits [178, 179]. Compared with direct thermal stimulation, exercise-induced HSP expression reflects a multifactorial stress response. Its magnitude is influenced by exercise modality, duration, intensity, environmental temperature, and the individual’s training status, resulting in relatively limited specificity and controllability [180]. When bone mineral density is used as the primary outcome, exercise intervention studies typically require at least 6 months of follow-up, and treatment adherence has a substantial influence on efficacy. Accordingly, stable clinical benefits generally require sustained long-term participation [181].

#### Precision control of HSPs expression via gene and cell therapies

As central regulators of stress responses in bone cells, HSPs control key pathways involved in bone remodeling, and their modulation may help restore skeletal homeostasis. Accordingly, precise regulation of HSP expression through gene- and cell-based therapies represents a promising emerging therapeutic strategy. Using lentiviral vectors to overexpress HSPB5, researchers showed that it binds directly to β-catenin, prevents its ubiquitination and degradation, thereby stabilizing β-catenin and augmenting Wnt signaling, which constitutes the core mechanism by which it promotes osteogenic differentiation [70]. This approach is currently widely used in cellular and animal experiments.

Small interfering RNA (siRNA) can selectively silence genes involved in pathological skeletal remodeling. Loss-of-function studies using siRNA-mediated CRYAB silencing have further confirmed the positive role of CRYAB in the osteogenic differentiation of hBMSCs [70]. siRNA technology can now be used to modulate both anabolic and catabolic pathways in bone with high precision [182, 183]. However, its therapeutic application remains limited by rapid degradation, short circulation time, poor tissue penetration, and off-target effects. Delivery to bone cells is particularly challenging because bone tissue has limited vascular perfusion and restricted drug accessibility [184]. Recent advances in nanoparticle-based carriers, including lipid-based systems and mineral-coated particles, may help overcome these limitations [182, 185]. For example, delivery of RANK-targeting siRNA to osteoclast precursors using mesoporous nanoparticles reduced osteoclast formation by approximately 70%, downregulated osteoclast-related genes, and preserved bone mass in osteoporosis models [186].

Following the success of mRNA vaccines, messenger RNA-based therapeutics have attracted increasing attention. This approach delivers synthetic mRNA encoding therapeutic proteins into patient cells, allowing transient protein production and offering a new means of delivering osteogenic factors without permanent genetic modification. In one study, 7-methylguanosine-capped Runx2 mRNA was delivered via bone-targeted lipid nanoparticles, resulting in a significant enhancement of osteoblast activity and bone formation in aged osteoporotic mice [187]. Compared with protein-based therapy, mRNA therapeutics offer several advantages, including controllable expression duration, relatively low immunogenicity, and reduced need for prolonged administration. Nevertheless, important challenges remain, particularly in relation to molecular stability, targeted delivery, safety, and ethical considerations [188, 189]. At present, HSPs are not major direct targets in mRNA therapy, which continues to focus primarily on osteogenic and angiogenic factors such as BMP-2 [190], Runx2 [187] and VEGF [36].

Chimeric antigen receptor macrophages (CAR-Ms) are an emerging cell-therapy platform derived from CAR-T technology. Early clinical trials in oncology have demonstrated the feasibility and safety of CAR-M therapy [191]. This platform has particular relevance to bone metabolic disorders because bone marrow macrophages, often referred to as osteomacs, regulate both osteoclast and osteoblast lineages and coordinate inflammation-associated bone resorption and bone formation [192, 193]. In addition, HSPs interact closely with macrophages [48, 60, 106, 123, 164]. Macrophages possess several inherent advantages, including tissue infiltration and homing capacity, phagocytic activity, antigen presentation, and cytokine secretion. CAR engineering further endows them with antigen-specific recognition, making CAR-M a potentially valuable platform for precision cell therapy [191]. However, compared with oncology, ideal target antigens for accurate CAR-M recognition in bone metabolic disorders such as osteoporosis remain poorly defined [193, 194]，Moreover, the engineering and quality control of CAR-M products remain technically complex, and issues of cost and scalability may represent major barriers to clinical translation [195].

CRISPR-based gene editing has also been increasingly explored in bone-related research. The CRISPR/Cas9 system enables precise genomic modification and has the potential to permanently correct genetic determinants of skeletal disease. In rare monogenic skeletal disorders, such as osteogenesis imperfecta and hypophosphatasia, CRISPR/Cas9 has successfully corrected COL1A1 mutations in patient-derived induced pluripotent stem cells (iPSCs), thereby resolving the underlying molecular defect associated with osteogenesis imperfecta [196]. In more common skeletal diseases, including osteoporosis, CRISPR has been investigated mainly for the modulation of pathogenic pathways and the development of functional models [197, 198]. Safety remains the foremost concern, as off-target DNA cleavage and mosaic editing may pose long-term risks [199, 200]. Base editing and prime editing may offer safer alternatives by enabling upregulation or downregulation of skeletal genes without introducing large double-strand breaks [201]. Although no CRISPR-based therapies for skeletal disorders have yet entered clinical trials, rapid advances in gene editing for hematologic and liver diseases suggest that future applications in bone metabolic disorders may become achievable [202, 203].

Exosomes are critical intercellular mediators among bone cells; by delivering proteins and miRNAs, they regulate osteogenesis, osteoclastogenesis, and bone remodeling, thereby maintaining bone homeostasis and representing a promising therapeutic platform for bone injury and disease [204]. Compared with β-tricalcium phosphate (β-TCP) scaffolds alone, BMSC-derived exosome/β-TCP composite scaffolds achieve superior repair of rat calvarial defects. Subsequent work showed that these exosomes are efficiently internalized by BMSCs, significantly enhancing proliferation, migration, and osteogenic differentiation [205]. Nevertheless, clinical translation faces substantial hurdles: exosomal functions in bone metabolism depend strongly on the donor cell type and its physiological/pathological microenvironment; moreover, isolation and purification, targeting control, and therapeutic cargo loading remain challenging [206].

Taken together, current approaches for targeting HSPs in osteoarthritis encompass multiple intervention modalities, including small molecules, biologics, physical therapies, and gene- or cell-based strategies. Although these approaches differ in mechanism and stage of development, each offers distinct therapeutic opportunities and translational challenges. For clarity, their major features are comparatively summarized in Table 3, which also provides a framework for the following discussion on translational prospects.

**Table 3:** Comparative evaluation of HSP-Targeted therapeutic strategies for OP: mechanisms, advantages, limitations, and translational feasibility

Intervention Category	Representative Agents/Platforms	Main Mechanism of Action	Application Model	Main Therapeutic Outcomes	Advantages	Limitations	Translational Feasibility	References
Small Molecule HSP90 Inhibitors	Broad-spectrum HSP90 inhibitors (e.g., 17-AAG) and HSP90β-selective inhibitors (e.g., KUNB31)	Inhibit HSP90’s ATPase activity, induce degradation of its client proteins, and block multiple pathogenic signaling pathways.	Tested in cell experiments and animal models; some inhibitors have entered clinical trials or are used for other diseases.	Enhances bone formation, inhibits bone resorption, and reverses bone loss.	Well-established development foundation with combined multi-pathway effects; isoform-selective inhibitors offer higher specificity and lower toxicity, with better safety and prospects.	Broad-spectrum inhibitors act on multiple HSP isoforms and their client proteins, which may disrupt bone homeostasis and cause side effects.	Translational feasibility is moderate. The clinical use of similar drugs provides a precedent, but issues such as bone-targeted delivery and reduction of side effects still need to be addressed to improve its feasibility in osteoporosis treatment.	[116, 122] [139, 143]
Small Molecule HSP Activators	Thiolutin (a thiol antibiotic compound)	Activates cellular stress responses to induce HSPB1 phosphorylation and HSP70 expression, enhance chaperone activity, stabilize the cytoskeleton, and inhibit mitochondria-mediated apoptosis, thereby promoting osteogenic differentiation and cell survival.	In vitro culture models of osteoblasts and BMSCs	Osteogenic phenotype is markedly enhanced: osteogenic marker expression and mineralization capacity are increased, with reduced apoptosis under stress conditions.	Rapid onset of action, producing significant biological effects even at low concentrations; unique mechanism of action, for example promoting osteogenic differentiation by stimulating HSP phosphorylation.	Exhibits pronounced dose-dependent toxicity, with high doses being highly cytotoxic, which limits clinical use; additionally, some compounds have low water solubility and bioavailability, and their sources are rare and hard to mass-produce.	Translational feasibility is low. Currently, toxicity and delivery issues remain unresolved; although strategies like prodrug design and bone-targeted conjugation have been proposed to improve this, there is still a considerable gap before safe and effective clinical application can be achieved.	[62] [144–147]
Natural Product Modulators	Flavonoids (e.g., luteolin), polyphenols (e.g., EGCG), and triterpenoids (e.g., triptolide)	Multi-target regulation of HSPs and related pathways: luteolin induces HSP40 and enhances RUNX2; EGCG enhances antioxidant activity via HSP27, promoting osteogenesis and inhibiting bone resorption; triterpenoids downregulate HSP90 and upregulate VEGF/Flk1 signaling to promote bone angiogenesis.	Mainly validated in cell and animal models, with some compounds progressing to clinical studies; EGCG inhibits osteoclastogenesis and reduces bone loss in ovariectomized osteoporotic rats.	Enhances bone formation and reduces inflammation and bone resorption, improving bone mineral density and bone mass in osteoporotic animal models.	High safety profile and wide availability, suitable for long-term use; also possesses antioxidant, anti-inflammatory, and other multiple effects, contributing to an overall regulation of the bone microenvironment.	Relatively mild efficacy and generally low bioavailability in vivo; individual metabolic differences may affect efficacy, and positive results in animal studies have not consistently translated to clinical success.	Translational feasibility is moderate, with a good safety profile and some evidence supporting bone protective effects, but the standalone efficacy is limited. Enhancing targeting via nanodelivery systems or structural modifications, or combining with other strategies, is needed to fully realize its translational potential.	[91, 125] [148–162]
Biologics	Neutralizing anti-HSP70 antibody	Neutralizes extracellular HSP70 (eHSP70) and blocks the immune “danger” signals it mediates via receptors such as TLR4/CD14, thereby reducing the release of inflammatory cytokines and the activation of osteoclasts.	Validated in animal disease models, but not yet in osteoporosis. In doxorubicin-induced heart failure mice, the neutralizing antibody reduced eHSP70, decreased neutrophil infiltration, increased M2 macrophages, and improved inflammation and cardiac function.	Significantly attenuates inflammatory responses and improves the function of damaged tissues; theoretically, it could also mitigate chronic inflammatory reactions in the bone microenvironment and prevent excessive osteoclast activation.	Clear target with effects confined to the extracellular space, thus not affecting the normal functions of intracellular HSP; therapeutic outcomes can also be monitored via eHSP levels in body fluids.	Long-term systemic neutralization of HSP70 may impair the body’s responses to infections and tissue injuries; additionally, there is no method to deliver the antibody specifically to bone tissue, and its efficacy in osteoporosis models has not yet been demonstrated.	Translational feasibility is moderate. Its clear mechanism of action and the efficacy observed in preliminary animal studies make it a promising novel immunomodulatory strategy. However, bone-targeted delivery methods need to be developed to reduce systemic side effects, and further validation of its safety and efficacy in osteoporosis models is required.	[106] [163–167]
Physical Therapies	Low-intensity pulsed ultrasound (LIPUS); pulsed electromagnetic fields (PEMF); mild heat stimulation	Uses non-invasive physical stress to induce upregulation of endogenous HSPs (primarily HSP70 and HSP90), activating osteogenic signaling pathways (including the BMP–Smad pathway) and inhibiting oxidative stress and inflammatory responses, thereby accelerating bone tissue repair.	Supported by cell and animal studies, with some modalities in clinical use. LIPUS promotes fracture healing and enhances osteogenesis and mineralization; PEMF relieves osteoporotic pain and stimulates bone formation; mild heat stimulation has mainly been validated ex vivo.	Can increase bone formation and mineralization, promote healing of fractures or bone defects, and alleviate osteoporosis-related pain; some of these physical modalities have been used clinically for bone repair and have shown preliminary efficacy.	Non-invasive with high safety, some methods are regulator-approved and in clinical use; can serve as adjuncts for long-term management of bone health.	Efficacy depends on strict parameter settings and requires frequent, sustained stimulation; the HSP-inducing effect is short-lived, necessitating daily treatment and thus poor long-term compliance; the induction mechanism involves multiple factors, resulting in relatively weak specificity and controllability.	Translational feasibility is high. The techniques are well-established with clinical experience and recognized safety, and can be directly used to aid in the prevention and treatment of osteoporosis. However, patients need to adhere to long-term therapy to achieve sustained benefits, and in practice these modalities serve more as adjunct improvements rather than a fundamental cure.	[58, 62] [100, 113] [161–183]
Gene/Cell Therapies	HSP gene modulation (e.g., CRYAB overexpression), siRNA/mRNA-based regulation, MSC-derived exosomes, and emerging engineered cell platforms	Precisely modulate HSP-related or bone-remodeling pathways at the gene/cell level: CRYAB overexpression stabilizes β-catenin and enhances Wnt signaling to promote osteogenesis; siRNA enables selective silencing of pathogenic targets; mRNA therapeutics provide transient expression of osteogenic factors without permanent genome modification; CAR-M exploits macrophage homing, phagocytosis, and antigen recognition for precision immunocellular therapy; MSC-derived exosomes deliver proteins/miRNAs to enhance osteogenesis and bone repair.	Mainly validated in cell experiments and animal models; early translational experience exists in related biomedical fields for mRNA therapeutics, CAR-M, and gene-editing platforms, but not yet as mature osteoporosis therapies.	Promotes osteogenesis and mineralization, inhibits osteoclastogenesis in selected settings, preserves bone mass, and improves bone defect repair/regeneration.	High specificity and programmability; enables precise pathway-level intervention; some platforms have bone-targeting or natural homing potential; mRNA avoids permanent genomic modification; exosomes and macrophage-based carriers may improve delivery efficiency and biocompatibility.	Rapid degradation and off-target effects for siRNA; stability, targeted delivery, and safety issues for mRNA; unclear target antigens, manufacturing complexity, cost, and scalability limitations for CAR-M; donor heterogeneity, purification, targeting control, and cargo-loading challenges for exosomes; long-term safety and regulatory issues remain unresolved.	Low-to-moderate. Despite strong precision and transformative potential, most approaches remain at an early preclinical or proof-of-concept stage in osteoporosis. Future progress will depend on bone-targeted delivery systems, improved safety control, scalable manufacturing, and clearer disease-specific targets.	[70] [182–206]

**References**: Numbers in brackets correspond to reference list entries. **Intervention category:** Indicates the major classes of HSP-targeted therapeutic strategies for osteoporosis. **Main mechanism of action:** Summarizes how each strategy modulates HSP-related pathways, bone remodeling, or the bone immune microenvironment. **Application model:** Refers to the experimental setting in which the strategy has been evaluated, including in vitro studies, animal models, or clinically related contexts. **Main therapeutic outcomes:** Summarizes the principal biological effects, such as promotion of osteogenesis, inhibition of osteoclastogenesis, suppression of inflammation, or alleviation of bone loss. **Advantages and limitations:** Reflect the major strengths and barriers of each strategy, including specificity, safety, bioavailability, delivery, and off-target effects. **Translational feasibility:** Reflects the current stage of evidence and the major challenges for clinical implementation

### Translational prospects of HSPs

Within the molecular networks that drive the onset and progression of OP, HSPs not only fulfill their canonical role as molecular chaperones but also act as regulators of immune homeostasis, actively participating in the dynamic remodeling of the bone immune microenvironment. As synthesized in this review, HSPs maintain protein homeostasis in OBs, OCs, and BMSCs and thereby modulate stress signaling and cell-fate decisions. Importantly, HSP functions are markedly dual: intracellular HSPs sustain protein folding and metabolic homeostasis—exerting anti-apoptotic, pro-osteogenic, and anti-inflammatory effects—whereas eHSPs, acting as DAMPs, can trigger immune inflammation and potentiate osteoclast activity. This inside-out role transition spans the continuum from molecular disequilibrium to structural deterioration in OP, providing a new mechanistic lens on bone immune dysregulation.

**Fig. 3:**
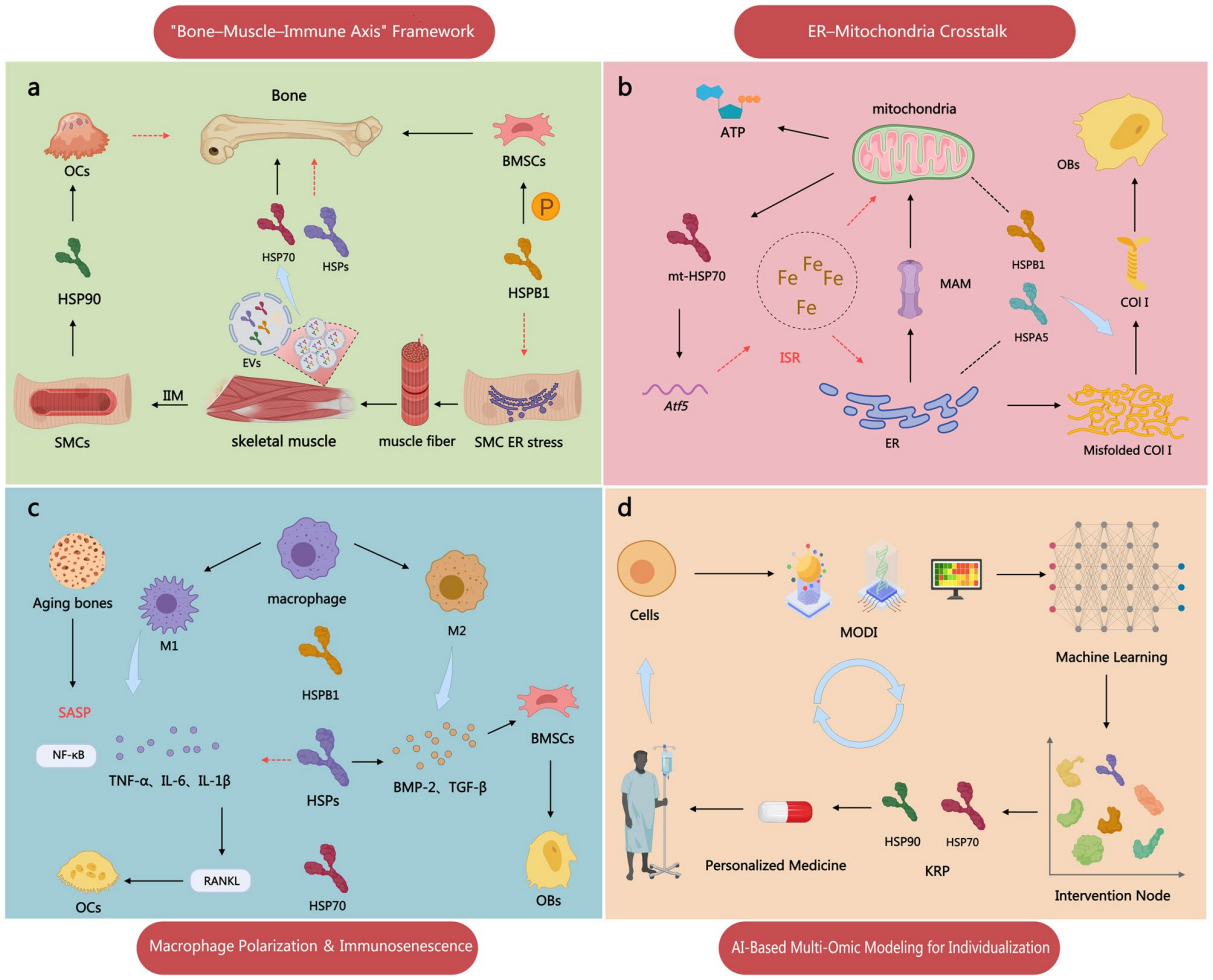
Conceptual framework of HSP-Mediated osteoimmune regulation in osteoporosis. This figure outlines four key aspects of HSPs’ systemic research: (**a**) The “Bone-Muscle-Immunity Axis” Framework. Osteoporosis and muscle atrophy share a common pathological basis, and bone and skeletal muscle are interconnected through EVs. Muscle-derived HSP70 promotes osteogenic differentiation of BMSCs, while HSPB1 stabilizes the mitochondrial function of myofibrils and phosphorylates BMSCs, acting as a link within the bone-muscle axis. In pathological conditions such as idiopathic myositis, HSP90 secreted by smooth muscle cells can aberrantly activate OC differentiation. (**b**) “Endoplasmic Reticulum-Mitochondria” Crosstalk. Iron overload triggers an ISR between the endoplasmic reticulum and mitochondria, inducing compensatory expression of HSPB1 and HSPA5, which assist in the refolding of misfolded proteins such as COLI to restore osteogenic function and maintain mitochondrial energy metabolism homeostasis. Concurrently, mitochondrial HSP70 suppresses excessive stress through feedback inhibition via the upregulation of the *Atf5* transcription factor. (**c**) Regulatory Strategies for Macrophage Polarization in the Context of Immunosenescence. Aged bone secretes SASP factors, including IL-6, TNF-α, and RANKL, which promote osteoclast differentiation by activating RANKL signaling. Macrophage differentiation into M1 and M2 subtypes secretes corresponding cytokines to regulate bone metabolism. HSPB1, HSP70, and other heat shock proteins play a role in this process by inhibiting SASP secretion and osteoclastogenesis, while promoting osteogenic differentiation of BMSCs. (**d**) Personalized Regulation of “Heat Shock Response-Bone Immunity” Using AI and Multi-Omics Modeling. Integrating multi-omics data from patients with bone loss, machine learning identifies patient-specific intervention nodes within the “Heat Shock Response-Bone Immunity” network. Based on this, targeted strategies for key regulatory proteins such as HSP70 and HSP90 are developed, combined with dynamic monitoring to implement a “prediction-intervention-validation” cycle for personalized precision medicine. Atf5: Activating Transcription Factor 5; BMP2: Bone Morphogenetic Protein 2; BMSCs: bone marrow mesenchymal stem cells; Col I: Collagen I; ER: Endoplasmic Reticulum; EVs: Extracellular Vesicles; HSPs: Heat shock proteins; IIMI: Idiopathic Inflammatory Myopathy; ISR: Integrated Stress Response; KRP: Key Regulatory Proteins; MAM: Mitochondria-Associated Membranes; MODI: Multi-Omics Data Integration; OBs: osteoblasts; OCs: osteoclasts; RANKL: Receptor Activator of Nuclear Factor-κB Ligand; Runx2: Runt-related transcription factor 2; SASP: senescence-associated secretory phenotype; SMCs: Smooth Muscle Cells; TGF-β: Transforming Growth Factor Beta; TNF-α: Tumor Necrosis Factor Alpha. (the figure was created in Medpeer)

Building on this foundation, the preceding sections delineate diverse, HSP-targeted intervention strategies spanning small-molecule agents, biologics, physical modalities, and gene–cell therapies. Research on HSPs has progressed from basic biology to an applications-oriented, translational exploration stage. However, the large and functionally pleiotropic HSP family necessitates isoform-specific, tissue-contextual, and immune-milieu–aware precision control during clinical translation, so as to avoid broad-spectrum modulation that perturbs bone remodeling or elicits adverse immune responses. As shown in Fig. 3, HSP-focused investigations are shifting from single-molecule analyses toward systems-level integration.

#### Building an integrated “bone–muscle–immune axis” framework

The link between OP and muscle wasting has garnered increasing attention, particularly through interrogation of their shared pathophysiological mechanisms. Skeletal muscle and bone communicate bidirectionally via neuroendocrine and immune signals; consequently, bone and muscle metabolism intersect to shape musculoskeletal function [207]. Heat shock proteins play central roles in bone-muscle crosstalk. In skeletal muscle, HSPB1 preserves myofiber contractile function by suppressing endoplasmic reticulum stress [208]. It also acts as a signaling molecule that promotes the osteogenic differentiation of hBMSCs [33]. In idiopathic inflammatory myopathy, HSP90 levels are elevated together with proinflammatory cytokines [209]. In parallel, HSP90β deficiency impairs osteoclast differentiation and disrupts bone remodeling [210]. Skeletal muscle-derived extracellular vesicles (EVs) provide an additional mechanism of communication. After entering the circulation and reaching the bone marrow, these EVs can be internalized by BMSCs and deliver glycolytic enzymes that enhance glucose metabolism and osteogenic potential. They are also enriched in chaperone proteins such as HSP70, which may further support bone formation [211]. Collectively, these findings indicate that HSPs may serve as key stress-response hubs linking bone, muscle, and immune networks. However, existing studies have focused mainly on individual tissues or single molecules, and a systematic translational framework is still lacking. Advancing the bone-muscle-immune axis from a conceptual model to a clinically useful paradigm will therefore require a staged and integrated research strategy.

This research strategy can be organized into five major components. The first is phenotypic characterization in human populations combined with analysis of HSP associations. Clinical cohorts of patients with osteoporosis accompanied by sarcopenia or impaired muscle function should be established. Key measurements should include bone mineral density, bone microarchitecture, muscle mass, muscle strength, and HSP profiles in circulating blood and extracellular vesicles. Comparative analysis could then identify the HSP isoforms most strongly associated with the composite phenotype of low bone mass, low muscle strength, and high inflammation. Preliminary cohort studies have examined relationships among bone mineral density, muscle strength, and inflammatory factors, but HSP profiling has not yet been incorporated [212]. The second component is in vitro mechanistic validation and causal investigation. Co-culture and organoid models incorporating myocytes, BMSCs or osteoblasts, and immune cells should be developed to determine whether HSPB1, HSP70, HSP90, and related proteins function merely as accompanying stress markers or instead act as regulatory hubs that shape bone-muscle-immune crosstalk [210, 211, 213]. The third component is causal inference using multi-omics and genetic approaches. After candidate HSP molecules have been identified, Mendelian randomization may be applied to population genetic data to assess causal associations between HSP-related variants and bone or muscle phenotypes. In parallel, single-cell RNA sequencing could be used to define differences in HSP expression in immune cells from individuals with high versus low bone mass, while spatial transcriptomics could localize HSP-high cell populations within the bone microenvironment. Together, these approaches may help determine whether specific HSPs actively contribute to bone loss progression [214]. The fourth component is the development of composite biomarkers and patient stratification. This step would help identify patients with HSP-related osteoporosis subtypes and support the future selection of targeted interventions. The fifth and final component is early intervention assessment and clinical translation. Once high-risk subtypes have been identified, small-scale interventional studies could test whether modulation of key nodes within the bone-muscle-immune axis improves clinical outcomes. Positive findings could then justify larger randomized controlled trials and ultimately provide a basis for individualized, integrated treatment strategies for patients with osteoporosis complicated by sarcopenia.

Overall, this strategy appears feasible. In the short term, key targets could be identified by establishing cohorts of patients with osteoporosis and sarcopenia and profiling relevant HSPs. In the medium term, the functions of candidate HSPs could be validated in vitro. In the long term, integrated biomarkers may be developed to guide coordinated prevention and treatment. The main technical challenge lies in integrating multiple factors across different tissues. Nevertheless, the core methodologies required for this work, including cohort follow-up, co-culture and organoid systems, and single-cell omics, are already well established, making this a practical and implementable translational pathway.

#### Focusing on ER–mitochondria crosstalk and the regulation of Osteocellular energy Metabolism

Crosstalk between endoplasmic reticulum stress and mitochondrial dysfunction is increasingly recognized as a key mechanism in the pathogenesis of osteoporosis. It integrates multiple pathological processes, including protein misfolding, calcium homeostasis imbalance, oxidative stress, and insufficient ATP production, into a coordinated pathogenic network. Current evidence has primarily demonstrated the involvement of this crosstalk in osteoblasts and BMSCs. In osteoblasts, procollagen misfolding can trigger an integrated stress response across organelles, accompanied by aberrant activation of the mt-HSP70/ATF5 axis. In addition, metabolic stressors such as iron overload can further promote mitochondrial fission, impair respiratory function, and amplify apoptotic signaling, thereby worsening defects in bone formation [101, 105, 215]. Under basal conditions, HSPB1 is imported into the mitochondrial intermembrane space, where it safeguards mitochondrial protein folding; loss of HSPB1 leads to organelle swelling and impaired respiratory function [216]. In iron-overloaded OB models, ER-mitochondrial crosstalk induces mitochondrial fission and dysfunction [217], underscoring a tight link between ER-mitochondria communication and the regulation of osteocellular energy metabolism, with HSPs positioned as pivotal modulators.

Clinical research should begin with stratification by pathological subtype and screening for HSP-associated targets. Across major clinical phenotypes, including postmenopausal osteoporosis and glucocorticoid-induced bone loss, systematic assessment of the structural integrity of endoplasmic reticulum–mitochondria contact sites, activation of individual unfolded protein response branches, mitochondrial membrane potential, oxygen consumption rate, and ATP production is needed to identify the HSP subtypes most closely associated with impaired bone formation [105, 215]. During mechanistic validation, primary hBMSCs, OBs, bone organoids, and conditional genetically modified animal models should be combined with ultrastructural imaging, Seahorse metabolic analysis, and stable isotope tracing to distinguish correlative stress markers from actionable pathogenic targets [101]. Because bone biopsy is invasive and has limited clinical applicability, priority should be given to developing a composite biomarker platform that integrates peripheral blood-derived HSPs, ER stress-related proteins, markers of mitochondrial injury, bone turnover markers, bone microarchitectural parameters, and fracture outcomes. Such a platform could generate a multidimensional signature of disease activity [218–220]. At the intervention stage, broad-spectrum HSP modulation should not be advanced directly to clinical application. Instead, efforts should focus on bone-targeted delivery, controlled correction of ER stress, restoration of mitochondrial homeostasis, and precise modulation of HSP function, while simultaneously evaluating extraskeletal metabolic effects, cardiac safety, and immunological safety in animal studies [221, 222]. Once sufficient preclinical and translational evidence has been established, biomarker-enriched early-phase clinical studies can be initiated. These studies should preferentially enroll patient subgroups with well-defined phenotypes characterized by disturbed energy metabolism or activated ER stress, and should use early pharmacodynamic responses, bone turnover markers, and improvements in bone microstructure as interim endpoints rather than relying solely on bone mineral density changes [223–225].

Basic research has shown that coupling between endoplasmic reticulum stress and mitochondrial dysfunction impairs osteogenesis, with HSPB1 and the mtHSP70/ATF5 axis among the key mediators. The translational potential of this pathway is moderate. Cell-based assays and animal models are readily implemented, but conversion of these findings into effective therapies remains challenging. The proposed strategies, including development of a peripheral blood-based multicomponent “energy metabolism–bone” signature and bone-targeted delivery systems, are supported by a reasonable practical foundation. Candidate agents, such as mitochondria-targeted antioxidants and small molecules that modulate protein folding, are already under investigation. However, major challenges remain in achieving precise cross-organelle regulation and maintaining safety. Overall, with improved mechanistic stratification and biomarker-based prescreening, this field has the potential to support small-scale clinical validation over the medium to long term and to improve interventions for metabolic stress-associated osteoporosis.

#### Strategies to regulate macrophage polarization in the context of immunosenescence

With aging, immunosenescence characterized by low-grade chronic inflammation and dysregulated immune phenotypes disrupts the balance between bone formation and resorption, culminating in progressive bone loss [226]. Senescent bone and bone-marrow cells exhibit a senescence-associated secretory phenotype (SASP)-including IL-6, TNF-α, and RANKL-that reprograms the local immune landscape and skews remodeling toward enhanced osteoclastogenesis and impaired osteogenesis [227]. Macrophage polarization is tightly linked to OP: M1 macrophages produce TNF-α, IL-6, and IL-1β, driving OC differentiation/activation and chronic bone resorption, whereas M2 macrophages secrete osteogenic factors (e.g., BMP-2, TGF-β) that promote BMSC-to-OB commitment, enhance mineralization, and restrain OC activity. Therapeutically steering M1 to M2 polarization thus represents a safe and potentially effective strategy for OP [192]. Aged macrophages may exhibit a state of dual dysregulation, characterized by elevated basal inflammatory activity but impaired specialized functions, including phagocytosis and activation of adaptive immunity [228]. Emerging evidence indicates that, in the context of immunosenescence, altered macrophage function and polarization are important drivers of chronic inflammation and age-related disorders, including inflammatory osteoporosis [229].

HSPs show stage-specific and phenotype-specific expression patterns during the differentiation of human monocytes into macrophages and during macrophage polarization. Specific HSP family members, including HSPB1, HSPA2, and HSPBAP1, may play critical roles in macrophage maturation, polarization, and immune responses, highlighting their potential importance in immune-related diseases [230]. Upregulation of HSP70 markedly suppresses the expression of the proinflammatory mediators TNF-α and IL-6, as well as NF-κB activation, in LPS-stimulated macrophages; this anti-inflammatory effect is abolished following HSP70 deletion [231]. Under inflammatory conditions, dysregulation of factors such as RANKL may subsequently promote osteoclast activation and osteoporotic bone loss [232]. Collectively, these findings suggest that HSPs are essential for maintaining macrophage polarization balance and may also delay immunosenescence by modulating immunometabolism and cellular stress responses. Modulation of HSP expression has already shown beneficial effects in cellular and animal models by alleviating markers of immunosenescence and osteoporosis, thereby supporting further target identification and mechanistic investigation.

Animal models of immunosenescence are now well established and include naturally aged models and D-galactose-induced models [233, 234]. These systems enable multidimensional evaluation of macrophage polarization and function under immunosenescent conditions through immune phenotyping, functional assays, and assessment of senescence-associated biomarkers [235, 236] Previous studies targeting macrophage polarization have shown that local IL-4 administration in aged rats with bone defects shifts macrophages from the M1 to the M2 phenotype, significantly suppresses excessive local activation of the NLRP3 inflammasome, and reduces inflammatory mediators such as IL-1β. These findings support the feasibility of enhancing anti-inflammatory macrophage signaling to improve tissue repair in aging populations [235].

Strategies aimed at regulating macrophage function through HSP targeting remain at the preclinical stage. Even if appropriate targets and therapeutic agents are identified, they will require validation through multiple stages of development. Denosumab provides an instructive precedent. Its development originated from fundamental discoveries involving the RANK/RANKL/OPG axis. After this osteoimmunological pathway was established as a therapeutic target, denosumab was developed to neutralize RANKL and inhibit osteoclast activity through immune-mediated mechanisms [237, 238]. In clinical development, phase I studies demonstrated favorable safety and dose-dependent suppression of bone turnover [239], phase II trials showed significant increases in bone mineral density [240], and large phase III trials confirmed substantial reductions in vertebral and hip fracture risk in high-risk older adults [241]. In 2010, denosumab was approved by the U.S. Food and Drug Administration for the treatment of postmenopausal osteoporosis, marking a milestone in the clinical translation of an immune-pathway target for skeletal disease. Similarly, clinical translation of HSP-targeted strategies designed to restore immune homeostasis and rebalance macrophage polarization will require stepwise evidence generation and carefully designed trials. With guidance from successful precedents and careful consideration of the unique characteristics of older populations, this field may ultimately yield new therapeutic approaches based on the biology of immunosenescence.

Chronic inflammation associated with immune aging, including secretion of SASP factors, together with polarization of macrophages toward the M1 phenotype, is a major contributor to bone loss. HSP70 and related molecules can suppress macrophage-derived proinflammatory mediators, and restoration of macrophage polarization balance has already shown therapeutic potential in experimental models of osteoporosis. The clinical feasibility of this strategy appears relatively high. The success of denosumab has already demonstrated that osteoporosis can be effectively targeted through immune-pathway intervention. In parallel, aging models and macrophage-targeting approaches, such as IL-4-induced M2 polarization, are well established and have shown efficacy in promoting bone repair. If suitable HSP-related targets can be identified, small-molecule or antibody-based therapies may be advanced into clinical testing relatively rapidly. However, because immune status in older patients is highly complex, stepwise clinical validation remains essential to ensure both safety and efficacy.

#### Leveraging artificial intelligence and multiomics modeling for personalized control of the heat shock response and bone immune state

Advances in biotechnology and computational methods have enabled the integration of large-scale omics data with artificial intelligence (AI), accelerating the transition of osteoporosis research from empirical approaches to precision medicine. Increasing evidence shows that integrative multi-omics analysis can uncover molecular subtypes and pathogenic pathways that are difficult to identify using conventional methods. In one study, multidimensional datasets, including transcriptomic and proteomic profiles, were analyzed using feature-selection approaches such as random forest (RF) and support vector machine (SVM), leading to identification of the FOXO3–SIRT1 regulatory axis as a key oxidative stress-related biomarker and potential therapeutic target in osteoporosis [242]. AI-based models have also been applied to investigate the immune-related molecular basis of osteoporosis. Using machine-learning methods, including LASSO regression and SVM recursive feature elimination, researchers identified five immune-related genes, including *Ccr5*, *Iapp*, and *Ifna4*, from peripheral blood transcriptomic data. These markers effectively distinguished individuals with high and low bone mineral density and supported the development of predictive models for osteoporosis risk [243]. Prospective multi-omics studies based on large longitudinal cohorts have also begun to emerge. Yuan et al. generated the first multi-omics atlas of osteoporosis using serial samples from a cohort of 366 individuals and applied an interpretable AI framework based on Deep Latent Space Fusion (DLSF). Their analysis identified two molecular subtypes among Chinese patients with osteoporosis. These subtypes exhibited differential responses in bone mineral density to calcium supplementation over 2 years, and differences in fracture risk became evident during 4 years of follow-up. These findings indicate that integration of multimodal omics signals, including genetic, transcriptomic, and metabolic data, can identify clinically relevant patient subgroups and facilitate the development of individualized intervention strategies [244]. Overall, the integration of multi-omics and AI has shown considerable potential for identifying key regulators of the osteoimmune network and refining molecular subtype classification.

However, individualized strategies for assessing and targeting the HSP-mediated heat shock response–osteoimmune state in osteoporosis remain lacking. Regulation of HSP expression is a highly coordinated, multilayered process encompassing transcriptional, posttranscriptional, translational, and degradative controls, enabling precise and efficient tuning under cellular stress. Single cell and single molecule imaging approaches reveal spatial dynamics of *Hsp* gene expression, such as mRNA localization and movement of transcription sites, and indicate that HSP mRNAs can be “tagged” cotranscriptionally to prioritize cytoplasmic translation [245]. Single cell RNA sequencing has been used to profile the bone immune microenvironment in one patient with osteoporotic vertebral compression fracture and one with vertebral ischemic necrosis, resolving cellular compositions, differentiation trajectories, and cell to cell interactions. These data reconstruct the progression from osteoporotic vertebral compression fracture to vertebral ischemic necrosis, characterized by BMSC depletion, immune suppression, and heightened osteoclast activity tipping remodeling toward resorption, and they enable dynamic readouts of immune state and outcomes in bone loss disorders [246]. A recent study of the HSP90 inhibitor 17-AAG in prostate cancer established a unified paradigm that links mechanism, reference analyte, and prediction; using GFFS based machine learning combined with Boolean minimization, the HSR was compressed into a protein logic signature, enabling accurate prediction of 17-AAG efficacy [131]. This paradigm can be ported to bone immune disorders by using single cell multiomics as input to prioritize three to five core regulators from the HSP90 and HSP70 systems, derive a binary signature of the bone immune state, guide personalized selection of HSP inhibitors, and through dynamic monitoring create a closed loop of prediction, intervention, and validation, thereby delivering a computable and clinically actionable plan for individualized control of the HSR in osteoporotic bone loss.

Current research priorities should focus first on integrating multi-omics datasets to identify key regulatory networks. The scope of multi-omics analysis should be broadened to include metabolomic and microbiome data, which should be analyzed alongside genomic and proteomic information to clarify interactions among metabolic, immune, and skeletal pathways in osteoporosis [247]. The next priority is the development and validation of individualized models. Building on the key networks identified, and informed by the integrated “mechanism–biomarker–prediction” Boolean network framework developed by Shin et al. in prostate cancer [131], mathematical models should be established to characterize individual heat shock response states. Emerging technologies should be incorporated to enhance model complexity and biological resolution, while new algorithms and computational tools should be further developed. These may include graph-based approaches for mining multi-omics relationships and reinforcement learning methods for optimizing individualized intervention strategies. In drug development, AI models could be used to predict patient responses to existing osteoporosis therapies, such as bisphosphonates and romosozumab, and may also facilitate the design of novel agents through generative models targeting pathways identified by these computational analyses [248]. Most importantly, the application of multi-omics-based AI should be extended into clinical trials and prospective interventional studies. High-risk populations identified by these models should be enrolled in intervention studies to determine whether model-guided personalized treatment can improve clinically meaningful outcomes, particularly fracture reduction. Despite the substantial promise of AI and multi-omics in osteoporosis and osteoimmunology research, implementation remains challenging. One major obstacle is data integration in the context of limited sample size. Multi-omics datasets are high dimensional and highly heterogeneous, and data generated across platforms are often affected by batch effects and technical noise. As a result, extracting robust cross-omics features from relatively small cohorts remains difficult [249]. A second challenge is model interpretability and biological validation. Although complex models, including deep learning approaches, often achieve high predictive performance, they are frequently difficult to interpret. Clinical application, however, requires transparent and explainable decision-making. Accordingly, key biomarkers and model predictions must be validated experimentally, for example through functional studies assessing the effects of candidate genes on osteogenesis or osteoclastogenesis, to support their mechanistic plausibility [250]. Clinical translation and integration into routine decision-making also remain significant barriers. Even a well-performing model must be incorporated effectively into clinical workflows to be useful in practice. Clinicians need simple, intuitive tools, such as structured reports and decision-support systems, to translate model outputs into actionable decisions [248]. Once these challenges are overcome, AI-driven decision-support tools may provide reliable guidance for clinicians and enable practical precision intervention strategies.

**Fig. 4:**
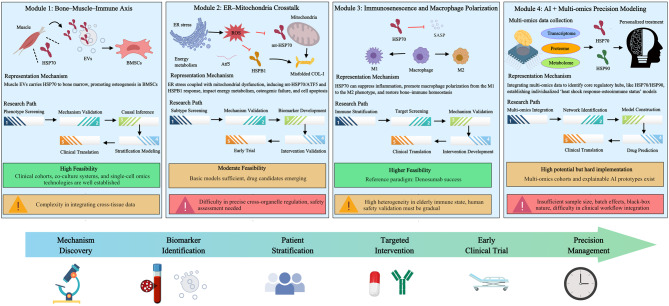
Translational roadmap for HSP-Based precision intervention in osteoporosis. This figure provides a systems-level overview of four prioritized directions for future HSP-centered research in osteoporosis. Module 1, the bone-muscle-immune axis, highlights that skeletal muscle may communicate with the bone marrow niche through extracellular vesicles carrying HSP70 and related stress signals, thereby modulating the osteogenic differentiation of BMSCs and suggesting a shared cross-tissue regulatory network linking osteoporosis and sarcopenia. Module 2, ER-mitochondria crosstalk, emphasizes the interplay among ER stress, ROS accumulation, compensatory mt-HSP70/HSPB1 responses, and collagen I misfolding, indicating that HSPs may coordinate proteostasis, bioenergetic homeostasis, and osteoblast survival during bone formation failure. Module 3, immunosenescence and macrophage polarization, illustrates that HSP70 may suppress SASP-related inflammatory signaling and promote macrophage polarization from the M1 to the M2 phenotype, thereby restoring bone-immune homeostasis and representing a comparatively feasible immunomodulatory strategy. Module 4, AI-assisted multi-omics precision modeling, proposes integration of transcriptomic, proteomic, and metabolomic data to identify key regulatory hubs such as HSP70 and HSP90 and to establish individualized predictive and interventional models, reflecting a future direction toward precision medicine in osteoporosis. The lower arrow further summarizes the staged translational trajectory of the field, extending from mechanism discovery and biomarker identification to patient stratification, targeted intervention, early clinical trials, and ultimately precision management. The figure also indicates the relative feasibility and major implementation barriers of each module, underscoring the transition of HSP research from mechanistic exploration to stratified, testable, and clinically translatable application. EVs, extracellular vesicles; BMSCs, bone marrow mesenchymal stem cells; ER, endoplasmic reticulum; ROS, reactive oxygen species; mt-HSP70, mitochondrial heat shock protein 70; COL-I, collagen I; SASP, senescence-associated secretory phenotype; M1/M2, pro-inflammatory/anti-inflammatory macrophage polarization states. (the figure was created in Medpeer)

Taken together, these emerging priorities support a staged translational framework for HSP-centered research in OP. As illustrated in Fig. 4, the field is moving beyond mechanistic exploration toward biomarker discovery, patient stratification, targeted intervention, early clinical validation, and ultimately precision management. In contrast to Fig. 3, which highlights the conceptual framework of HSP-mediated osteoimmune regulation, Fig. 4 provides a more practice-oriented roadmap by integrating specific research modules, feasibility considerations, and translational challenges.

## Conclusions

Overall, research on HSPs is at a pivotal inflection point, transitioning from molecular function dissection to systems oriented intervention strategies. Future work should integrate the three dimensions of protein homeostasis, bioenergetics, and immune signaling, using interdisciplinary methods such as AI analytics and systems biology to achieve quantitative and individualized control of HSP regulation. As this framework matures, therapies that remodel HSP homeostasis could become a new paradigm for precise intervention in OP and its complications, providing a robust theoretical basis for improving bone quality, preventing fractures, and slowing aging related disturbances of bone metabolism.

## Data Availability

The datasets used and/or analysed during the current study are available from the published literature cited in the reference list. No new experimental data were generated for this study.

## Abbreviations

ADSCs: adipose-derived stem cells
AGEs: Advanced glycation end products
AI: artificial intelligence
AIS: adolescent idiopathic scoliosis
ALP: alkaline phosphatase
Atf5: Activating Transcription Factor 5
ATP: adenosine triphosphate
BMD: bone mineral density
BMP2: Bone Morphogenetic Protein 2
BMSCs: bone marrow mesenchymal stem cells
CAR-Ms: chimeric antigen receptor macrophages
CCs: chondrocytes
Col I: Collagen I
CRISPR: clustered regularly interspaced short palindromic repeats
C-SRC: Cellular Src tyrosine kinase
CTD: C-terminal domain
DAMPs: damage-associated molecular patterns
DLSF: Deep Latent Space Fusion
ECM: extracellular matrix
ER: Endoplasmic Reticulum
ERK: Extracellular Signal-Regulated Kinase
EVs: Extracellular Vesicles
FDA: Food and Drug Administration
FTH1: ferritin heavy chain 1
GR: glucocorticoid receptor
GRP: glucose-regulated protein
hBMSCs: human bone marrow mesenchymal stem cells
HIF-1α: hypoxia-inducible factor-1 alpha
HSEs: heat shock elements
HSFs: heat shock transcription factors
HSPs: Heat shock proteins
HSR: heat shock response
IFN-γ: interferon-gamma
IGF-1: insulin-like growth factor 1
IIMI: Idiopathic Inflammatory Myopathy
IL-1β: interleukin-1 beta
IL-4: interleukin-4
IL-6: interleukin-6
iPSCs: induced pluripotent stem cells
ISR: Integrated Stress Response
KRP: Key Regulatory Proteins
LASSO: least absolute shrinkage and selection operator
LIPUS: low-intensity pulsed ultrasound
MAM: Mitochondria-Associated Membranes
MAPK: mitogen-activated protein kinase
MD: middle domain
MODI: Multi-Omics Data Integration
mt-HSP70: mitochondrial heat shock protein 70
NEF: nucleotide exchange factor
NF-κB: nuclear factor kappa-light-chain-enhancer of activated B cells
NFATc1: nuclear factor of activated T cells 1
NK: natural killer
NTD: N-terminal domain
OBs: osteoblasts
OCs: osteoclasts
OCN: osteocalcin
OCYs: Osteocytes
OP: Osteoporosis
OPG: osteoprotegerin
OPN: osteopontin
PEMF: pulsed electromagnetic field
PPARγ: peroxisome proliferator–activated receptor γ
RANKL: Receptor Activator of Nuclear Factor-κB Ligand
ROS: reactive oxygen species
Runx2: Runt-related transcription factor 2
SASP: senescence-associated secretory phenotype
siRNA: small interfering RNA
SMCs: Smooth Muscle Cells
SOX9: SRY-box transcription factor 9
SPP1: secreted phosphoprotein 1
SREC1: Sialic acid-binding immunoglobulin-like lectin 1
TGF-β: Transforming Growth Factor Beta
TLR: Toll-like Receptor
TNF-α: Tumor Necrosis Factor Alpha
TRAP: tartrate-resistant acid phosphatase
TUNEL: terminal deoxynucleotidyl transferase dUTP nick end labeling
VEGF: Vascular Endothelial Growth Factor
β-TCP: β-tricalcium phosphate
